# Genome-Wide Analysis, Classification, Evolution, and Expression Analysis of the Cytochrome P450 93 Family in Land Plants

**DOI:** 10.1371/journal.pone.0165020

**Published:** 2016-10-19

**Authors:** Hai Du, Feng Ran, Hong-Li Dong, Jing Wen, Jia-Na Li, Zhe Liang

**Affiliations:** 1 College of Agronomy and Biotechnology, Southwest University, Chongqing, 400716, China; 2 Department of Biological Sciences, National University of Singapore, 117543, Singapore, Singapore; National Taiwan University, TAIWAN

## Abstract

Cytochrome P450 93 family (CYP93) belonging to the cytochrome P450 superfamily plays important roles in diverse plant processes. However, no previous studies have investigated the evolution and expression of the members of this family. In this study, we performed comprehensive genome-wide analysis to identify CYP93 genes in 60 green plants. In all, 214 CYP93 proteins were identified; they were specifically found in flowering plants and could be classified into ten subfamilies—CYP93A–K, with the last two being identified first. CYP93A is the ancestor that was derived in flowering plants, and the remaining showed lineage-specific distribution—CYP93B and CYP93C are present in dicots; CYP93F is distributed only in Poaceae; CYP93G and CYP93J are monocot-specific; CYP93E is unique to legumes; CYP93H and CYP93K are only found in *Aquilegia coerulea*, and CYP93D is Brassicaceae-specific. Each subfamily generally has conserved gene numbers, structures, and characteristics, indicating functional conservation during evolution. Synonymous nucleotide substitution (*d*_N_/*d*_S_) analysis showed that CYP93 genes are under strong negative selection. Comparative expression analyses of CYP93 genes in dicots and monocots revealed that they are preferentially expressed in the roots and tend to be induced by biotic and/or abiotic stresses, in accordance with their well-known functions in plant secondary biosynthesis.

## Introduction

Cytochrome P450 monooxygenases (P450s) are widely distributed in eukaryotes; they form a large and diverse class of enzymes, with more than 35,000 members [1–2] (http://drnelson.uthsc.edu/P450.count.April.6.2016.PPTX). P450s are involved in numerous biosynthetic and xenobiotic pathways such as the assimilation of carbon sources, detoxification of xenobiotics, and synthesis of secondary metabolites [2–3]. They have relatively low sequence identity among different organisms (such as plants and animals); however, they show a common overall topology and tridimensional fold. Moreover, all P450s share four common characteristic regions—the proline-rich membrane hinge; I-helix involved in oxygen binding; K-helix (ExxR); and “PERF” consensus, which consists of the ERR triad involved in locking the heme pocket into position to ensure the stabilization of the conserved core structure [4–5].

In plants, the cytochrome (CYP) P450 superfamily is one of the largest gene families of enzyme proteins, and it includes 245 genes in *Arabidopsis* [6–7] and 332 full-length genes and 378 pseudogenes in soybean [8]. Plant P450s were originally classified into two main types—the A-type and non-A type [9–10]. Based on the numerous available genome sequences, researchers subsequently re-classified them into 11 clans. The A-type was grouped as the CYP71 clan, whereas the non-A type was subdivided into 10 clans—CYP51, CYP72, CYP74, CYP85, CYP86, CYP97, CYP710, CYP711, CYP727, and CYP746 [1,3,10]—according to a common nomenclature system [11]. However, new clans might emerge in the future as more lineages of plants are sequenced.

Plant P450s participate in various biochemical pathways leading to the production of primary and secondary metabolites [3]. For instance, most structural genes in the flavonoid and/or isoflavonoid biosynthesis pathways are CYP P450 genes such as *C4H* (*CYP73*), *F3′5′H* (*CYP75A*), and *IFS* (*CYP93C*) [12]. Notably, the diversification of P450s had a significant biochemical impact on the emergence of new metabolic pathways during the evolutionary process of land plants. A typical example is cytochrome P450 93C (CYP93C), which is mainly identified in legumes and is the first key enzyme involved in the metabolism of the legume-specific isoflavonoid biosynthesis pathway [12–16]. Therefore, CYP93C is of considerable interest in the genetic and metabolic engineering fields for its important roles in plant defense and human health, since it enhances the dietary intake of isoflavonoids and thus facilitates disease prevention. Similarly, several other members such as CYP93B, CYP93E, and CYP93G are known to play important roles as plant secondary metabolites, such as flavone [17–22] and triterpenoid saponin biosynthesis [23]. However, unlike the large number of P450 gene families in angiosperms, only a few members of CYP93 genes have been functionally characterized.

To date, genome-wide analysis of P450 super gene family has been performed in many plant species, based on the available genome sequences, such as *Arabidopsis* [10,24], *Oryza sativa*, *Vitis vinifera* L., *Populus tremuloides* [25], and *Nelumbo nucifera* [26]. However, a similar comprehensive analysis of P450 proteins in a given gene family, such as CYP93 genes, is still lacking. Thus, the gene structures, evolutionary relationship, and expression patterns of CYP93 gene family in land plants is yet not known, and a complete survey and classification in plants from distinct evolutionary groups are necessary; such data could markedly enhance our understanding of the evolutionary history as well as functions of CYP93 genes as plant secondary metabolites. Therefore, in the present study, we investigated the evolutionary history of the CYP93 family across 60 plants (including 53 angiosperms). We performed structural and evolutionary analyses across different plant evolutionary lineages and assessed their origins, classification, evolutionary relationship, and expression. Our study extended the sequence and functional characteristics of CYP93 gene family in land plants and might facilitate future functional analysis.

## Material and Methods

### Sequence retrieval

To identify CYP93 genes in plant genomes, we performed BLASTP searches of the sequenced plant genomes in the Phytozome (http://www.phytozome.net/) [27] and PGDD (http://chibba.pgml.uga.edu/duplication/) [28] databases by using representative CYP93 proteins such as CYP93A1, CYP93E1, and CYP93C1v2 as queries (*p*-value <e^-10^). The detailed information of the representative sequences is listed in S1 Table. The species represented a broad range of plant lineages, from unicellular green algae to multicellular plants. We used a relatively uniform criterion [1,11] to collect CYP93 genes with high-quality sequences. Next, the candidates were sent to Dr. David Nelson for uniform nomenclature (David Nelson, P450 Nomenclature Committee, personal communication). The detailed information (i.e., accession numbers) regarding the CYP93 sequences is listed in S2 Table.

### Sequence alignment and phylogenetic analyses

The CYP93 sequences were aligned using MAFFT version 7 [29] with the G-INS-i algorithm, followed by manual editing in MEGA 6.06 [30]. In our subsequent analyses, we only included positions that were unambiguously aligned. The neighbor joining (NJ) phylogeny was performed using MEGA 6.06 with 1000 replicates; we used the p-distance model and pairwise deletion. Similarly, a maximum likelihood (ML) tree was constructed using MEGA v6.06, with 100 replicates and Poisson distribution.

### Gene expression analysis

The *Arabidopsis*, soybean, maize, and rice microarray-based datasets (GEO accession numbers: GSE17883, GSE18518, GSE26198, GSE41125, GSE6901, GSE6908, GSE14275, GSE35984, GSE19024, GSE33410, GSE9687, and GSE35427) were downloaded from the Plant Expression database (PLEXdb, http://www.plexdb.org/index.php) [31]. A hierarchical cluster was created using Cluster 3.0 [32] by using hierarchical clustering based on log2 transformed data of the normalized expression data and mean method and viewed using Java TreeView [33].

### Expression analysis of CYP93 genes in *Arabidopsis*, soybean, maize, and rice

The expression information of the CYP93 genes in *Arabidopsis*, soybean, maize, and rice was further confirmed by real-time PCR. Given the high homology of the candidates in a species, all gene-specific primers were designed to avoid false priming by creating gene-specific sites at the 3ʹ-terminal of each primer, leading to the amplification of 100–200-bp long products. The specific primers for the actin gene were used as an internal control for each species. The PCR primer sequences are shown in S3 Table.

The plant tissues (roots, stems, leaves, flowers, etc) of maize, rice, *Arabidopsis*, and soybean were harvested and ground in liquid nitrogen. Total RNA was extracted using Eastep^™^ total RNA Extraction kit, according to the manufacturer’s instructions, and treated with DNase I (Promega, USA). First-strand cDNA synthesis was performed using an oligo (dT) primer and 2 μg of total RNA in a 20-μl reaction volume, according to the manufacturer’s instructions for the M-MuLV RT kit (Takara Biotechnology, Japan). The real-time PCR thermocycling parameters were as follows: an initial denaturation for 3 min at 95°C, followed by 45 cycles of a denaturation step at 95°C for 15 s and an annealing step at 58°C for 20 s. The fluorescence was measured after the extension step by using the CFX Connect^™^ Real-Time System (Bio-Rad). After the thermocycling reaction, the melting step was performed from 65°C up to 95°C, with an increment of 0.5°C each 0.05 s. Each PCR pattern was independently verified in three replicate experiments. The specificity of primers used in this study was verified by cloning the PCR products into the pTA2-T vectors (TOYOBO, Japan), and then using them for sequencing (data not shown).

### Selection analysis

We performed selection analysis as described previously [34]. The cDNA sequences were aligned using MUSCLE in MEGA5.2.2 software [35] by using the codon model algorithm and were then loaded into HyPhy [36], along with the corresponding ML phylogenetic trees. The HyPhy batch file NucModelCompare.bf with a model rejection level of 0.0002 was used to establish the best fit of 203 general time-reversible (GTR) models of nucleotide substitution. The HyPhy batch file QuickSelectionDetection.bf was used to estimate site-by-site variations in nucleotide substitution rates.

The structure was modeled using the SWISS-MODEL [37]. Template search was performed using Protein BLAST. The best model 5e58.1.A was selected based on Q-Mean. PyMOL was used for protein structure analysis and prepare figures.

## Results

### Distribution of CYP93 genes across angiosperms

To identify candidate genes in angiosperms, we used the known CYP93 representative sequences as quires to search the 60 sequenced species in the Phytozome v10 (http://www.phytozome.net/) [27] and PGDD (http://chibba.pgml.uga.edu/duplication/) databases [28].

**Fig 1:**
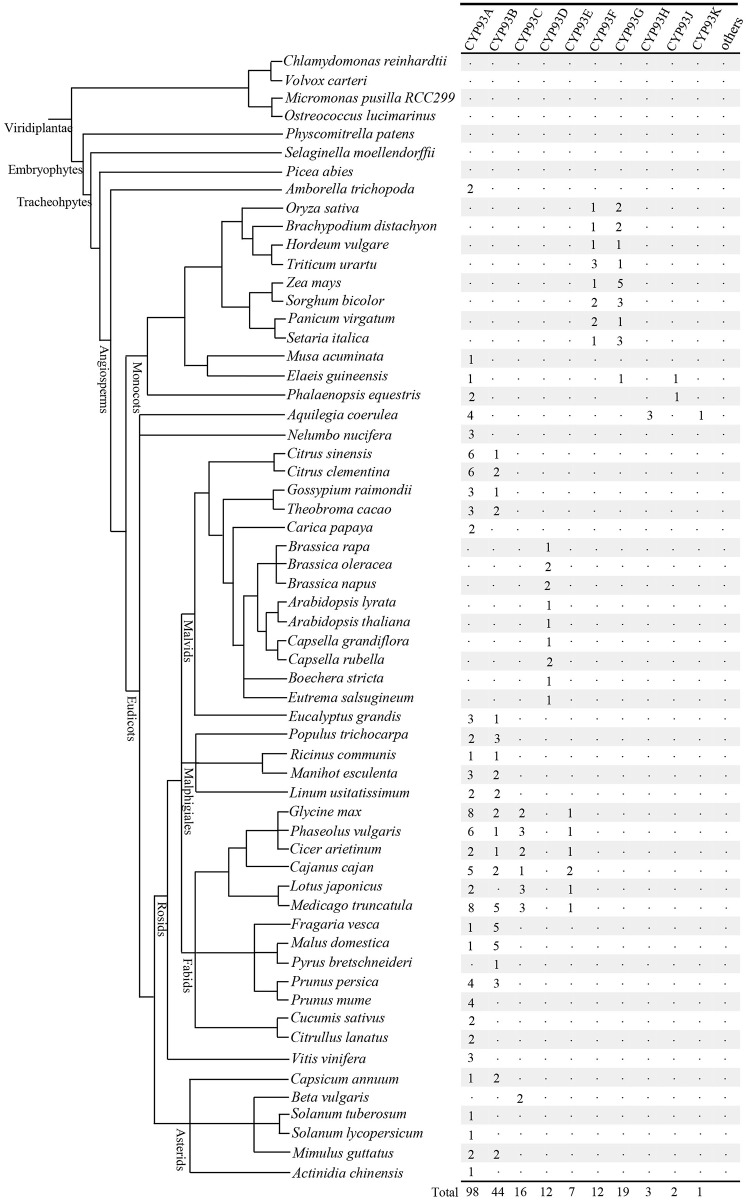
Phylogenetic relationships of the 60 species investigated in the present study. Phylogenetic relationships (branch lengths are arbitrary) among these species have been described previously (http://www.phytozome.net/). The total number of cytochrome P450 93 (CYP93) proteins identified in each genome is indicated on the right.

We identified 214 candidate genes in almost all the flowering plants among the 60 angiosperms investigated (Fig 1 and S2 Table). In contrast, we did not identify any CYP93 sequences in the non-flowering plant genomes, including chlorophyte algae (*Chlamydomonas reinhardtii*, *Volvox carteri*, *Micromonas pusilla RCC299*, and *Ostreococcus lucimarinus*), Bryophyta (*Physcomitrella patens*), lycophytes (*Selaginella moellendorffii*), or gymnosperms (*Picea abies*; Fig 1). To confirm these results, we then searched the NCBI database, including expressed sequence tags (ESTs), and did not find any CYP93s in the non-flowering species included in this database as well. Interestingly, we also did not identify any CYP93 candidates in the genome of *Beta vulgaris*, but found two partial sequences in Genebank that were previously reported; thus, we included them in our following analysis.

The CYP93 gene family was found to have a wide distribution in flowering plants. Since CYP93 proteins are present both in monocots and eudicots, the appearance of CYP93 genes was thought to predate the divergence of monocots and eudicots (Fig 1).

### Phylogenetic analysis of the CYP93 gene family

According to the common nomenclature system, P450s in the same family generally share at least 40% identity, and those in the same subfamily share at least 55% identity [11]. However, owing to gene duplication and shuffling, a straightforward nomenclature might be difficult; hence, family definition is recommended for integrating phylogeny and organization of genes [7].

Initially, we used an established nomenclature to group candidates by comparing their identity, which was performed by Dr. Nelson [11]. Our results showed that the amino acid sequences of all the CYP93 candidate proteins shared approximately 53% identity (S4 Table). The sequence identities among the CYP93A, CYP93B, CYP93C, CYP93D, CYP93E, CYP93F, CYP93G, CYP93H, and CYP93J proteins (CYP93K has only one member) were approximately 65%, 63%, 82%, 83%, 70%, 65%, 66%, 80%, and 47%, respectively. This result showed that the sequence homology in this gene family was highly conserved.

**Fig 2:**
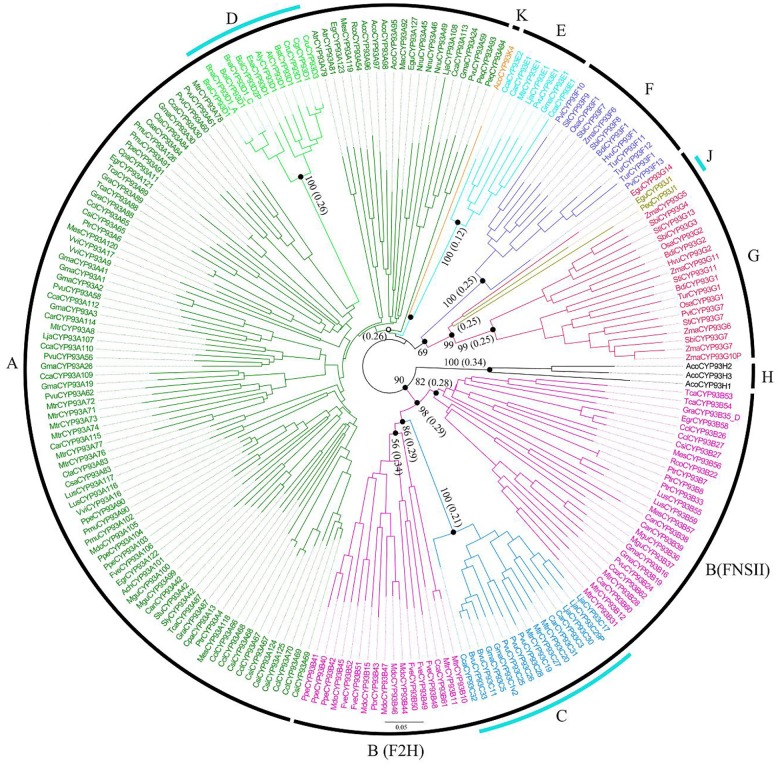
Phylogenetic tree and classification of 214 plant CYP93 proteins. The neighbor joining (NJ) tree includes 214 CYP93 proteins from 60 eukaryotes. Proteins are clustered into eight subfamilies (e.g., CYP93A). The colored lines and names symbolize the species to which the proteins in each clade belong. The black dots represent the major clades in the phylogenetic tree, and the corresponding bootstrap support values from 1000 replications are shown beside the black dots. Bootstrap values <50% are shown as black circles, and those >50% are shown as black dots in the phylogenetic tree. The numbers in brackets indicate the dN/dS value for each subfamily or branch. The information of species abbreviations used for the tree of Fig 2 is listed in S2 Table. The scale bar represents amino acid substitutions per site.

To elucidate the evolutionary relationships of this gene family in plants, we aligned the 214 candidate CYP93 proteins by using MAFFT software and constructed NJ and ML phylogenetic trees (Fig 2 and S1 Fig). Our results showed that the topologies of these two analyses were highly consistent. In our phylogenetic trees, the candidates from the same lineage tended to be clustered together, resulting in many lineage-specific subfamilies and/or clades. This might indicate that this gene family could have evolved or been lost in a specific lineage, following divergence. Moreover, the CYP93 proteins were not equally represented in different species, suggesting that they experienced duplications after the divergence (Fig 2).

In the phylogenetic tree, the CYP93 proteins were clustered into ten independent clades with high bootstrap support (except CYP93A), generally following the evolutionary order (Fig 2 and S2 Fig). Our dataset yielded several interesting findings. First, CYP93A is the largest subfamily, which includes genes from nearly all flowering plants (including monocots and dicots), except grasses and Brassicaceae, which formed some lineage-specific subfamilies instead. Second, CYP93B sequences also have a relatively wider distribution in plants and are present in many eudicot species, but not in Brassicaceae. Third, CYP93D sequences are distributed only in Brassicaceae and are embedded in the CYP93A subfamily, indicating that they might have originated from one or more gene duplications from ancient CYP93A subfamilies. Fourth, similar to CYP93D, CYP93C has members in all legumes and in *Beta vulgaris* and is embedded in the CYP93B subfamily, implying that it originated from CYP93B and was subsequently conserved during the evolution. Fifth, CYP93E sequences are specifically found in legumes. Sixth, the monocot CYP93 sequences were clustered into the CYP93F, CYP93G, and CYP93J branches; the CYP93F branch showed orthologous distribution only in grasses, whereas the CYP93G branch had members in grasses and *Elaeis guineensis*, indicating that it is older than CYP93F. Finally, the CYP93H and CYP93K sequences were distributed only in the basic dicot species *Aquilegia coerulea* (Figs 1 and 2).

Taken together, phylogenetic analysis revealed that the CYP93 genes were newly derived in flowering plants: an initial divergence from an ancient CYP93A clade by a gene duplication event during evolution, and a subsequent divergence via gene duplication in the CYP93A clade to yield the subfamilies CYP93B−CYP93K.

### Conserved characteristics of CYP93 sequences

**Fig 3:**
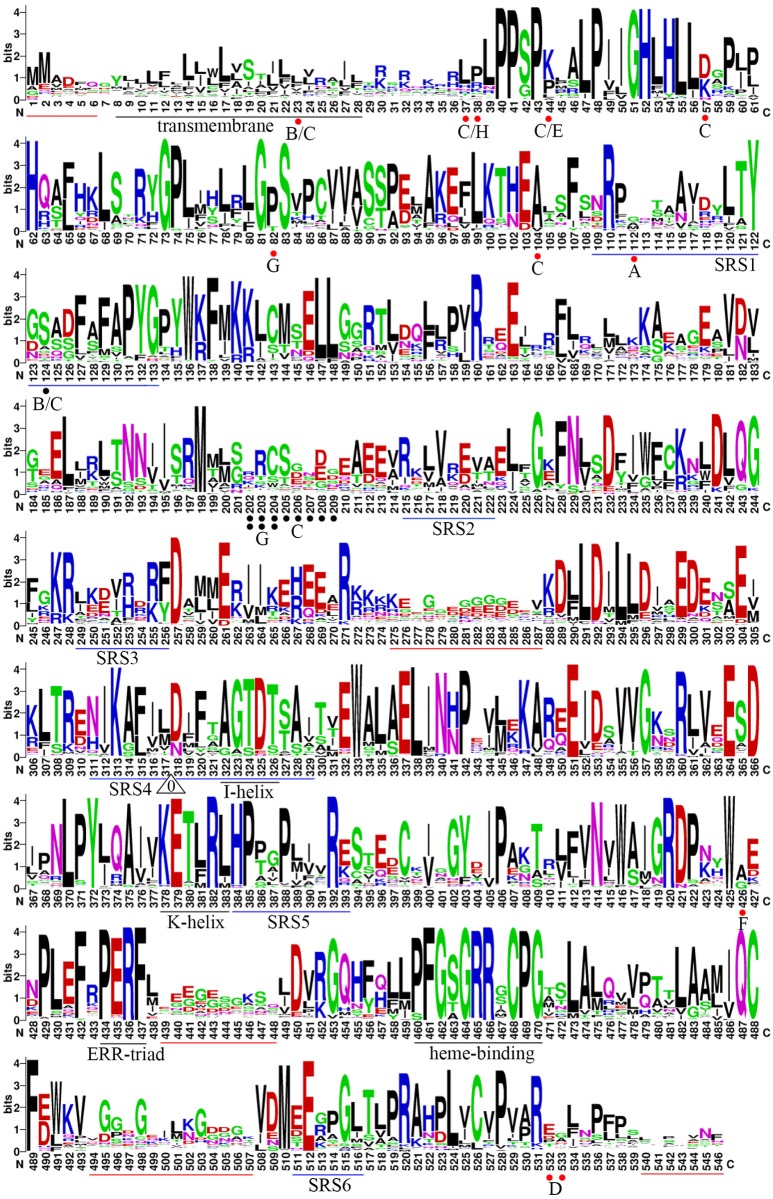
Sequence logos of the multiple alignments of the 214 CYP93 proteins in plants. The sequence logos of plant CYP93 proteins based on amino acid alignment using MAFFT are shown. The logos were generated using Weblogo. The bit score indicates the information content for each position in the sequence. The height of the letter designating the amino acid residue at each position represents the degree of conservation. The key conserved motifs are underlined; the red lines indicate the less conserved regions; the black ones, the P450 motifs; and the blue ones, the substrate recognition sites (SRSs). The white triangles indicate the conserved intron insertion location of plant CYP93 genes; the numbers within the triangles indicate the splicing phase of the intron (0 refers to phase 0). The red and black dots indicate the conserved amino acid insertion or deletion sites, respectively, in a given subfamily and/or clade; the number below each dot indicates the corresponding subfamily, i.e., B indicates the CYP93B subfamily.

The amino acid sequences of P450s are relatively diverse and have sequence identities as low as 20% [38]. However, the overall secondary and tertiary structures of P450s are highly conserved, because some primary regions/motifs such as the K-helix, PERF, and heme-binding domains, which are important to the secondary and tertiary structures, are conserved across eukaryotic P450s [3,20]. We assessed the sequence characteristics of the CYP93 gene family by conducting sequence alignment analysis (S2 Fig) and protein homology modeling (S3 Fig). The entire sequence of the CYP93 proteins based on our alignment analysis is shown in Fig 3.

**Fig 4:**
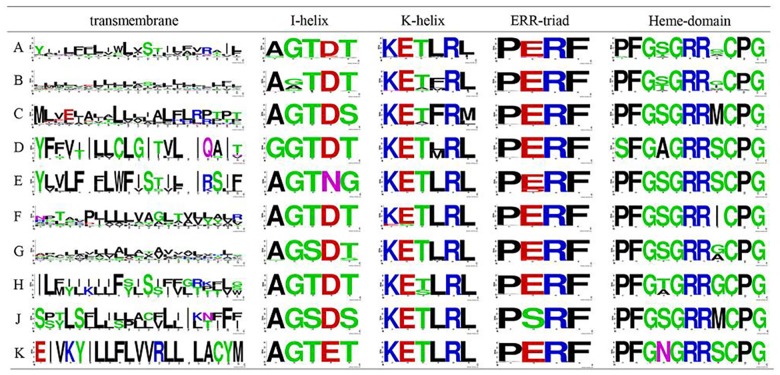
Architecture of conserved protein motifs in the eight subgroups of the plant CYP93 family. The sequence logos of the P450 transmembrane, I-helix, K-helix, PERF, and heme-binding motifs based on the amino acid alignments are shown. The bit score indicates the information content for each position in the sequence. A−K indicate subfamilies CYP93A−CYP93K.

One striking feature is that the overall protein modeling structures are very similar to each other (S3 Fig); thus, our detailed sequence analysis was mainly based on sequence alignment. With the exception of five regions having relatively low homology and variable sequence characteristics and lengths (especially the N- and C-terminal regions), the sequences of CYP93 proteins generally shared a high degree of similarity (Fig 3). Moreover, the sequences contained some highly conserved short amino acid motifs that are distributed throughout the coding regions (Fig 3). For example, consistent with the findings of previous studies [3,20], the four well-known P450 motifs—the PERF, K-helix, I-helix, and heme-binding domains—are generally relatively conserved across different subfamilies (>87% identity). However, the transmembrane domain shares only approximately 24% identity (Fig 4). In addition, with the exception of the commonly conserved residues, some clade- or subfamily-specific insertion or deletion sites are present. For instance, the sites at positions 57 and 104 are distributed only in the CYP93C subfamily, and the amino acids at positions 202–204 are missing in the CYP93G subfamily (Fig 3). Since these positions are located on the protein surface (S3 Fig), they might affect protein–protein interactions. Interestingly, we also found lineage-specific conservation of several amino acid substitutions at specific sites in the heme-binding and I-helix domains, compared to those in PERF and K-helix (Fig 4). Based on these findings, we speculated that most CYP93 genes are placed in a clear monophyletic clade and have subfamily-specific features. For example, the residues in the fourth and eighth sites of the heme-binding domain varied across different subfamilies—most CYP93A subfamily members have a conserved G/S residue in the eighth site, whereas those of the CYP93C−CYP93J subfamilies have conserved I, S, or M residues instead (Fig 4). These findings suggest that conserved residue substitutions in different subfamilies are key variants during evolution.

**Fig 5:**
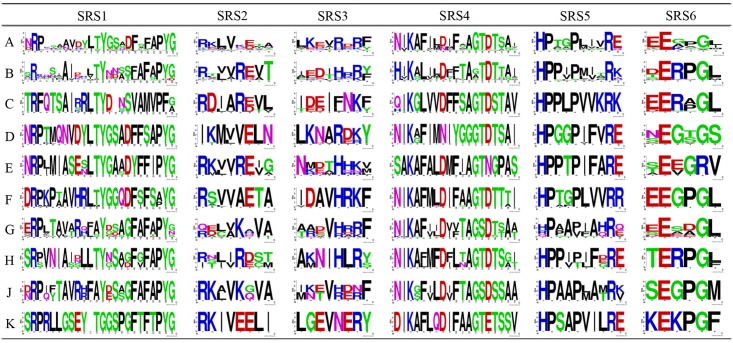
Weblogo of SRSs based on the amino acid alignments across the eight subgroups of the plant CYP93 family. The bit score indicates the information content for each position in the sequence. A−K indicate subfamilies CYP93A−CYP93K.

Despite considerable sequence variation between P450 enzymes, six substrate recognition sites (SRS1−SRS6) were identified as essential residues [39], and several key amino acid residues that affect substrate specificity are known to be found within these SRSs [40–42]. Thus, SRSs might be important for P450 binding and substrate recognition specificity (Fig 5). To investigate the sequence characteristics of these regions among the ten CYP93 subfamilies, we further compared the corresponding sequences. Our results showed that the overall identities among the SRS1, SRS2, SRS3, SRS4, SRS5, and SRS6 regions were approximately 62%, 50%, 39%, 70%, 64%, and 56%, respectively, indicating that these regions are less conserved compared to the four P450 motifs discussed above. Moreover, the residues in these six SRSs generally showed some obvious subfamily-specific site-substitutions, implying that they are involved in functional diversification among different CYP93 subfamilies. For instance, Ser 310 (326 in the present study), Leu 371 (387), and Lys 375 (391) were reported to be the key active-site residues for the CYP93C2 enzyme [13,43]. Ser 310 and Leu 371 are critical for substrate accommodation of the CYP93C2 enzyme in favor of hydrogen abstraction from C-3 of the flavanone molecule and in the presence of Lys 375, respectively. Lys 375 is responsible for aryl migration. Interestingly, the Ser 310 residue is located in the I-helix motif embedded in SRS4 and shows subfamily-specific substitutions (Fig 4). Similarly, Leu 371 and Lys 375 are located in SRS5, which also show conserved substitutions between different subfamilies (Fig 5). Therefore, these results confirmed that the SRS regions played an important role in the functional diversification of CYP93 families during evolution.

Furthermore, based on the available genome sequences (DNA and cDNA sequences), we analyzed the intron distributions in the encoding regions of the CYP93 genes. Our results revealed that 128 of the 160 analyzed CYP93 genes (80%) were disrupted by the highly conserved “M” intron [3,10], 26 genes (16%) had introns inserted at one or more sites, and only six genes (4%) were intron-less (Fig 3). Interestingly, most of the 26 genes having more than two introns had the conserved “M” intron site, whereas the remaining insertion sites were not conserved; this could be attributed to the inaccurate genome annotation among some species. Moreover, all the 26 multi-intron genes were CYP93A and CYP93B genes, whereas the six intron-less were CYP93G and CYP93F (S2 Table). Overall, with the exception of a few genes (4%), the majority of plant CYP93 genes (96%) have a highly conserved intron insertion position (Fig 3). Our results revealed that the splicing phase of the conserved intron was completely conserved to phase 0 (Fig 3) [44], indicating that the splicing mechanism of CYP93 genes was highly conserved during evolution. Considering that the main intron pattern is consistent with that of CYP712 and CYP705 family [10], these gene families could be thought to have a common ancestor.

Taken together, our findings suggest that the exon/intron structure of the CYP93 gene family was highly conserved during evolution (Fig 3); moreover, the SRSs generally show subfamily-specific amino acid substitutions at some sites (Fig 5), highlighting the nature of their functional diversification in different subfamilies.

### Role of selection in the CYP93 coding sequences

Comparing the dN and dS rates is a common method of determining selection pressures on coding regions. Commonly, a dN/dS value of 1 is used to indicate neutral selection, and values of <1 or >1 are used to indicate purifying and positive selection, respectively.

We investigated the influence of selective constraints on the CYP93 coding sequence. By globally fitting an evolutionary model, we calculated the ratios for each subfamily and/or clade (S5 Table). The dN/dS value of each subfamily or clade was found to be <1 in all groups, suggesting that strong purifying selection has been maintained across plants and implying that CYP93 genes are functionally conserved (Fig 2). At the individual codon level, most residues were under significant negative selection except several sites that were under positive selection; however, many sites were also under relaxed constraint. Consistent with sequence analysis, these relaxed constraint sites were mainly located in the regions with low homology and had variable lengths, and they were less frequent in the relatively conserved P450 domains and SRS regions (S5 Table).

### Expression analysis of CYP93 genes at different developmental stages

**Fig 6:**
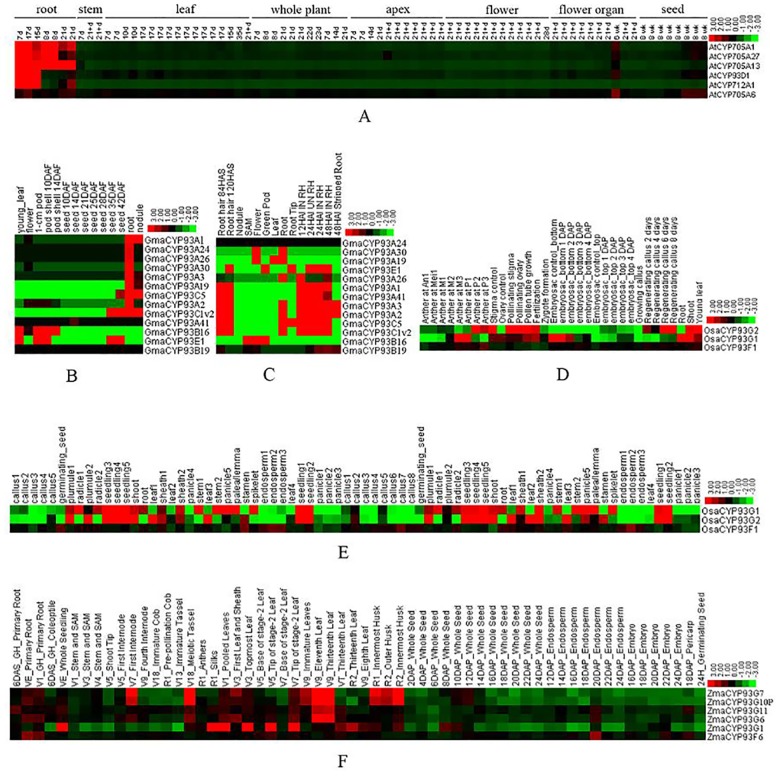
Expression profiles of CYP93 homologous genes in *Arabidopsis*, soybean, rice, and maize. (A) Expression profiles of the *AtCYP93D1* gene in *Arabidopsis*. (B) and (C) expression profiles of *GmCYP93* genes in soybean expression dataset1[46] and dataset2[47]. (D) and (E) expression profiles of *OsCYP93* genes in rice expression dataset1 (GSE14304) and dataset2 (GSE19024)[31]. (F) expression profiles of *ZmCYP93* genes in maize[48]. Color bar at the base represents log2 expression values.

To understand the expression profiles of CYP93 genes and elucidate their functions, we compared their expression profiles across two representative dicot and monocot plants, including *Arabidopsis*, soybean, maize, and rice (Fig 6).

Based on the AtGenExpress dataset [45], we showed that *AtCYP93D1* was strongly expressed in the roots and weakly expressed in the other tissues and/or organs (Fig 6A). Soybean dataset1 [46] results suggested that most CYP93A and CYP93C genes were preferentially expressed in the roots (Fig 6B), with the exception of *GmaCYP93A41* and *GmaCYP93B19* that were not expressed or very weakly expressed in all the tissues and organs investigated. Further, *GmaCYP93A2*, *GmaCYP93A24*, *GmaCYP93A3*, *GmaCYP93A19*, *GmaCYP93A26*, and *GmaCYP93A30* were expressed only in the roots; *GmaCYP93A1* was expressed in both the roots and nodules; *GmaCYP93C1v2* and *GmaCYP93C5* were strongly expressed in 35–42-day-old seeds, nodules, and roots, with the highest expression levels in roots; *GmaCYP93E1* was strongly expressed in young leaves, green pods, and 35–42-day-old seeds; and *GmCYP93B16* was expressed in early pods and young leaves. Similarly, the soybean dataset2 [47] suggested that the CYP93 genes were more strongly expressed in the roots and root hairs, except for *GmaCYP93A1* and *GmaCYP93B2*, which were not remarkably expressed in all the tissues investigated (Fig 6C): *GmaCYP93A1*, *GmaCYP93A2*, *GmaCYP93A3*, *GmaCYP93A26*, *GmaCYP93A41*, *GmaCYP93C1v2*, and *GmaCYP93C5* were mainly expressed in the roots and root hairs; *GmaCYP93A19* and *GmaCYP93A30* were strongly expressed in flowers, roots, and leaves; *GmaCYP93B16* was strongly expressed in the shoot apical meristem, flowers, and green pods; and *GmaCYP93E1* showed the strongest expression levels in the root tip, green pods, and root hairs. Taken together, our results suggested that CYP93 genes are mainly expressed in dicot roots, root hairs, and/or nodules, implying their conserved expression pattern during evolution.

In rice, we used two relatively comprehensive expression profile datasets from PLEXdb [31]. Dataset1 suggested that *OsaCYP93G1* and *OsaCYP93G*2 showed relatively wide expression profiles, with strong expression in the seedlings, shoots, roots, radicles, stems, spikelets, and sheaths (Fig 6D). Dataset2 indicated that *OsaCYP93G1* and *OsaCYP93G2* showed similar expression profiles, with the strongest expression levels in the roots, leaves, embryo, shoots, stigma, and callus (Fig 6E). *OsaCYP93F1* was constitutively and very weakly expressed in all the investigated tissues and organs (Fig 6D and 6E). In maize [48], *ZmaCYP93G7*, *ZmaCYP93G5*, *ZmaCYP93G11*, *ZmaCYP93G10P*, and *ZmaCYP93G6* were relatively strongly expressed in the roots, seedlings, tassels, leaves, and husks (Fig 6F). *ZmaCYP93F6* was constitutively expressed in all the investigated tissues and organs, with slightly stronger expression levels in the roots and embryo.

Taken together, our results showed that dicot CYP93 genes were preferentially expressed in the root tissues, whereas monocot CYP93 genes were strongly expressed in the roots and many other tissues and/or organs, indicating wider expression in monocots than in dicots.

### The expression pattern of plant CYP93 genes by real-time PCR

**Fig 7:**
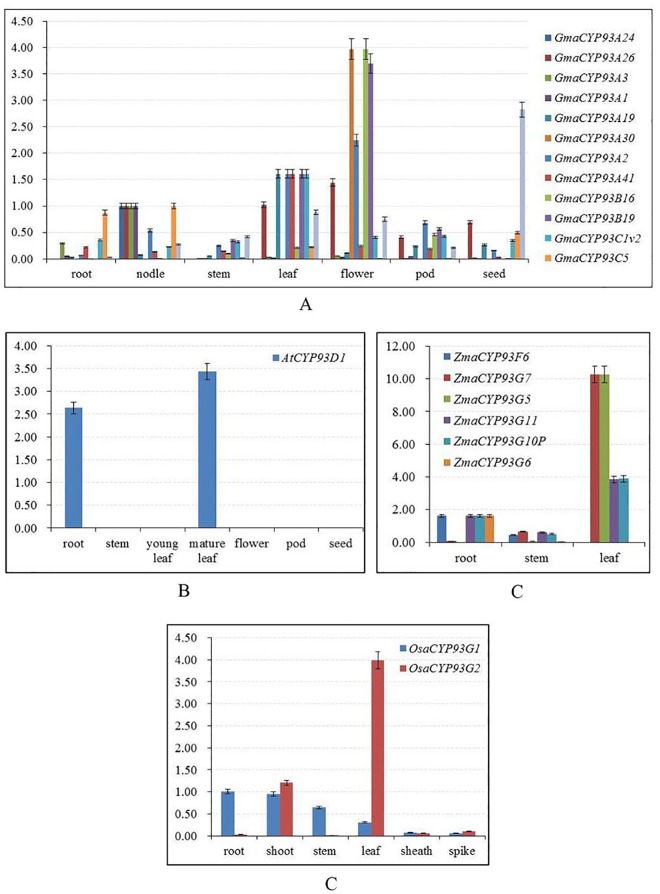
qRT-PCR analyses of the expression profiles of CYP93 homologous genes in *Arabidopsis*, soybean, rice, and maize. (A) Expression profiles of CYP93 genes in soybean. (B) Expression profiles of the *AtCYP93D1* gene in *Arabidopsis*. (C) Expression profiles of CYP93 genes in maize. (D) Expression profiles of CYP93 genes in rice.

Next, we analyzed the expression profiles of CYP93 gene family in *Arabidopsis*, soybean, maize and rice (Fig 7). The results showed that most of the CYP93 genes yielded positive qRT-PCR results, except *OsaCYP93F1*, which showed no expression signals (Fig 7D) and could be expressed at specific developmental stages or under special conditions.

In soybean, the *GmaCYP93* genes (*GmaCYP93A1*, *GmaCYPA2*, *GmaCYPA3*, *GmaCYPA19*, *GmaCYPA24*, *GmaCYPA26*, *GmaCYPA30*, *GmaCYPA41*, *GmaCYPB16*, *GmaCYPB19*, *GmaCYPC1v2*, and *GmaCYPC5*) showed high expression in the roots, stems, leaves, flowers, and seeds (Fig 7A). In *Arabidopsis*, the *AtCYP93D* showed relatively higher expression level in the roots and leaves (Fig 7B). In maize, *ZmaCYP93G5*, *ZmaCYP93G7*, *ZmaCYP93G11*, and *ZmaCYP93G10P* were highly expressed in the leaves, whereas *ZmaCYP93F6* and *ZmaCYP93G6* showed preferential expression in the roots (Fig 7C). In rice, *OsaCYP93G2* showed the highest transcript accumulation in the leaves, and *OsaCYP93G1* showed the highest accumulation in the roots and shoots (Fig 7D). In general, the expression patterns of closely grouped paralogs differed, suggesting that they had similar functions at different stages of plant development.

### Expression profiles of plant CYP93 genes in response to biotic and abiotic stresses

CYP93 genes are generally involved in plant secondary metabolism, which is important for plant defense responses, such as flavonoid and isoflavonoid compounds. To reveal the possible roles of CYP93 genes in stress responses, we analyzed their expression profiles in response to different biotic and abiotic stresses by using the publicly available global stress expression datasets in AtGenExpress [45] and PLEXdb [31].

**Fig 8:**
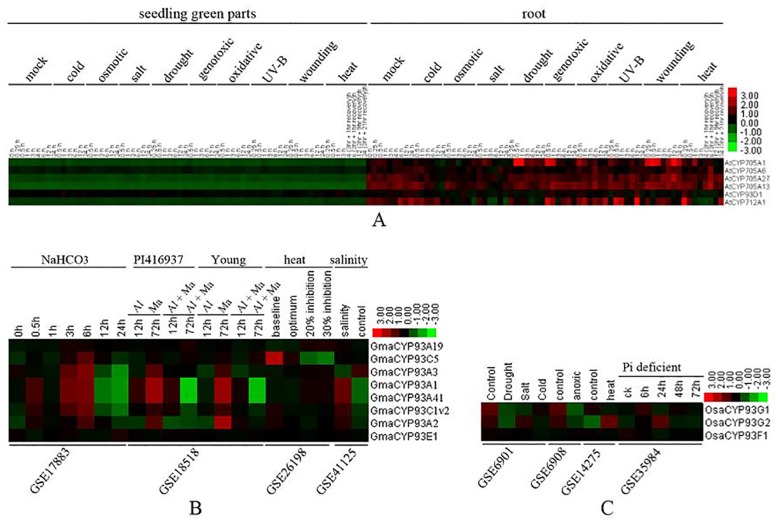
Expression profiles of plant CYP93 genes in response to abiotic stresses. (A) Expression profiles of *AtCYP93* and representative P450 genes in response to abiotic stresses. (B) Expression profiles of eight probe sets representing eight soybean CYP93 genes based on four microarray datasets of abiotic stresses. (C) Expression profiles of rice CYP93 genes based on four microarray datasets of abiotic stresses. Color bar at the base represents log2 expression values.

On the basis of the expression datasets in AtGenExpress [45], we first analyzed the expression patterns of *AtCYP93D1* under abiotic stress. *AtCYP93D1* was found to be preferentially expressed in the roots and only weakly expressed in the shoots (Fig 8A); it was strongly expressed in response to UV-B, cold, and heat treatments. In soybean, we identified eight probes—*GmaCYP93A1*, *GmaCYP93A2*, *GmaCYP93A3*, *GmaCYP93A19*, *GmaCYP93A41*, *GmaCYP93C1v2*, *GmaCYP93C5*, and *GmaCYP93E1*—corresponding to a single CYP93 gene (Fig 8B). However, we identified no probe set corresponding to the remaining five investigated genes. Hence, we used the eight target genes in our subsequent analysis. The expression levels of *GmaCYP93A1*, *GmaCYP93A2*, *GmaCYP93A3*, *GmaCYP93A41*, *GmaCYP93C1v2*, and *GmaCYP93C5* were markedly upregulated after NaHCO_3_ treatment, whereas those of *GmaCYP93A1*, *GmaCYP93A2*, *GmaCYP93A3*, and *GmaCYP93A41* were upregulated under conditions of magnesium stress, but suppressed in response to aluminum plus magnesium stress for 72 h. The expression levels of *GmaCYP93C5* and *GmaCYP93A3* were suppressed by heat and salinity stress, respectively; however, those of *GmaCYP93A1*, *GmaCYP93A2*, *GmaCYP93A41*, and *GmaCYP93C1v2* were upregulated in response to these stresses (Fig 8B). In rice, the expression level of *OsaCYP93G1* was suppressed by drought, salt, and cold, whereas that of *OsaCYP93G2* was suppressed by drought and cold. The expression level of *OsaCYP93G1* was suppressed whereas that of *OsaCYP93G2* was upregulated by heat. *OsaCYP93G1* and *OsaCYP93G2* were suppressed by anoxic stress, whereas they were upregulated under conditions of Pi deficiency for 6 h and 24 h, respectively. *OsaCYP93F1* showed weak and relatively consistent expression levels in response to all the investigated stresses (Fig 8C).

**Fig 9:**
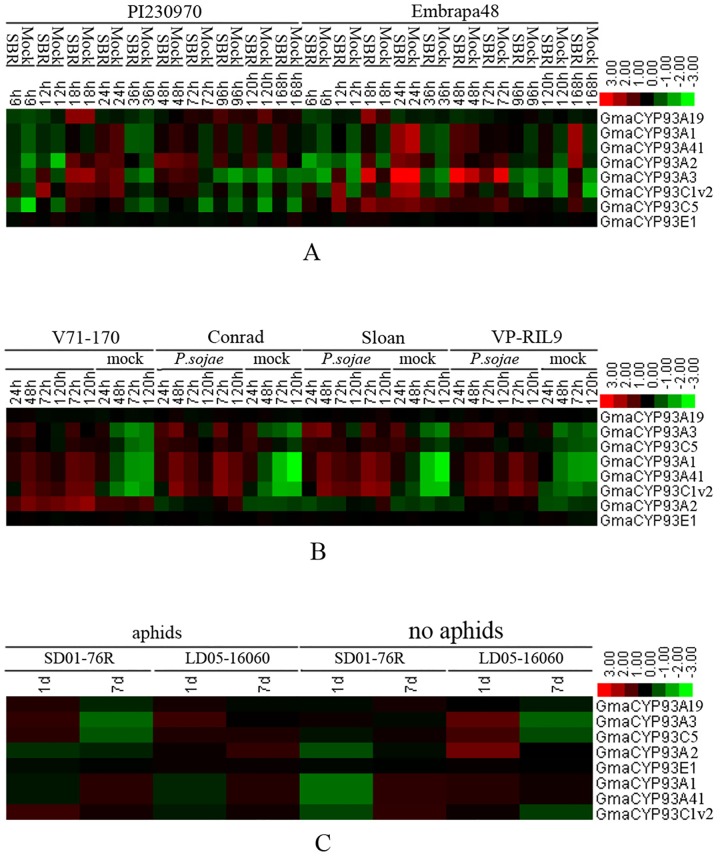
Expression profiles of *GmCYP93* genes in response to biotic stresses. (A) Expression profiles of GmCYP93 genes after infection with root-knot nematode (GSE33410). (B) Expression profiles of GmCYP93 genes after infection with *Phytophthora sojae* (GSE9687). (C) Expression profiles of GmCYP93 genes after aphid infestation (GSE35427). Color bar at the base represents log2 expression values.

Similarly, to elucidate the possible roles of CYP93 genes in biotic stress responses (pathogen and insect stress), we used the PLEXdb and AtGenExpress databases to determine their expression levels after exposure to different microbial and insect pathogens. However, the expression levels of *AtCYP93D1*, *OsaCYP93G1*, *OsaCYP93G2*, and *OsaCYP93F1* were not markedly upregulated after exposure to pathogen and insect stresses (data not shown). However, with the exception of *GmaCYP93E1*, the expression levels of soybean CYP93 genes were upregulated after exposure to rust disease (Fig 9A). Moreover, with the exception of *GmaCYP93A19* and *GmaCYP93E1*, the expression levels of soybean CYP93 genes were markedly upregulated after *Phytophthora sojae* infection, with comparable expression profiles across the four genotypes (Fig 9B). Similarly, except for *GmaCYP93A19* and *GmaCYP93E1*, the expression levels of soybean CYP93 genes were also upregulated after soybean aphid infestation (Fig 9C).

Taken together, our results suggested that the expression of plant CYP93 genes is upregulated in response to certain biotic and abiotic stresses, implying that the corresponding pathways or metabolites (e.g., isoflavonoids) that they regulate might be important for plant stress responses.

## Discussion

### Classification and evolution of CYP93 genes

In the present study, we analyzed the distributions of CYP93 genes in 60 plant genomes, including four green algae, one moss, one lycophyte, one gymnosperm, and 53 angiosperms. We generated a highly supported tree of candidate CYP93 proteins (Fig 2). The classical classification of the P450 family uses protein identity as the main criterion [1]. Our phylogenetic analysis tended to support this classification; however, some aspects are not in accordance with previous findings. In particular, several classified groups are not in parallel, e.g., CYP93D and CYP93C are embedded in CYP93A and CYP93B, respectively. These incongruities can be explained by the very recent origin of the new clade (subfamily CYP93C and CYP93D) in a specific lineage (such as Brassicaceae and Leguminosae) that share high sequence identity with the ancestor genes, and thus the phylogenetic tree lacked high resolution.

Based on the distributions and phylogeny, we postulate that all currently identified CYP93 genes are derived from a process involving gene duplications and diversification from a single progenitor molecule in the distant past of the last common ancestor of flowering plants. Considering that *Amborella trichopoda* contains two CYP93A candidates (Fig 1 and S2 Table), this gene family might have evolved after the split of angiosperms and gymnosperms and could have plant-specific flowering. However, since only the genome of *Picea abies* is currently available, this can be further verified only after more genomes can be used in this lineage. Thus, our results showed that the origin of the CYP93 family in land plants can be traced to the origin and early diversification of flowering plants. Moreover, CYP93A is likely the most ancient subfamily, because it has the broadest distribution across angiosperms and is the only CYP93 subfamily that occurs in both monocots and eudicots (Figs 1 and 2). The remaining CYP93 subfamilies are further subdivided into two groups—the monocot group (CYP93F, CYP93G, and CYP93J) and eudicot group (CYP93B, CYP93C, CYP93D, CYP93E, CYP93H, and CYP93K). Among these sub-families, subfamily CYP93B is relatively older and was present in the stem lineage of dicots (Fig 1). CYP93B is further subdivided into two clear monophyly clades, F2H and FNS II, each with high statistical support (82% and 56%; Fig 2). CYP93C is embedded in the CYP93B subfamily and forms a monophyly clade with a high bootstrap value (98%), which is more closely related to the F2H clade. These results supported that CYP93B and CYP93C share the same origin, where CYP93C is likely duplicated and/or retained from CYP93B after the divergence of legumes and other Fabids. This finding is strongly corroborated by independent evidence that CYP93B and CYP93C share the same substrate—(2S)-flavanone [13,14,18,20,44]. Monocots only contain CYP93G and/or CYP93F genes, and CYP93G might be older since its members are distributed in a relatively wider scope of species (Fig 1). Interestingly, several members of CYP93G were found to share the same substrate (flavanone) with CYP93B and CYP93C subfamilies [20–22]. This might suggest that the monocot and eudicot groups have a similar role in the conversion of flavanone to flavone.

To further elucidate the origin of CYP93 genes in P450 super gene family, we constructed a NJ phylogenetic tree by using the representative genes of each CYP93 subfamily and other plant P450 families, based on sequence alignment analysis (S4 Fig). Considering that the non-A type is very ancient and CYP51 protein is the only P450 family conserved across fungi, animals, and plants [11], we rooted the NJ tree on the representative of *Arabidopsis* CYP51G1. Our results showed that the CYP93 genes share a relatively high degree of sequence similarity with the CYP705 and CYP712 families (data not show) and cluster close to these two families in the phylogenetic tree, confirming that CYP93, CYP705, and CYP712 share a last common ancestor and CYP93 genes have evolved from CYP705 and/or CYP712 during the evolution [11].

### Functions of CYP93 genes

To date, some studies have focused on the narrow range of CYP93 genes, including CYP93A, CYP93B, CYP93C, CYP93E, and CYP93G (Table 1). Nearly all of the CYP93 family members functionally characterized thus far are involved in flavonoid/isoflavonoid biosynthesis, although the functions of CYP93D, CYP93F, CYP93H, CYP93K, and CYP93J are yet not known. For example, CYP93B is a known flavone synthase that produces flavones [17–18,20]; similarly, CYP93G1 and CYP93G2 in rice constitute flavone synthase II (FNS II) and catalyze the direct conversion of flavanones to flavones [20–22], and CYP93C is involved in isoflavonoid biosynthesis [13–16,49]. The exception is CYP93A and CYP93E subfamilies, where CYP93A is involved in pterocarpanoid phytoalexin biosynthesis in soybean [50], whereas CYP93E catalyzes the C-24 oxidation of the triterpene backbone in triterpenoid saponin biosynthesis [23].

**Table 1:** Summary of functionally characterized CYP93 genes in plants.

Subfamily	Name	Species	Biological Function	Reference
CYP93A	CYP93A1	soybean	pterocarpanoid phytoalexin biosynthesis	[50]
	CYP93A2	soybean	no known	[60]
CYP93B	CYP93B2	Gerbera	FNSII activity in flavone biosynthesis	[56]
	CYP93B1	licorice	F2H activities in licodione and flavone formation	[59]
	CYP90B10	*Medicago truncatula*	F2H activities that has a catalytic mechanism similar to FNS II	[17]
	CYP90B11	*Medicago truncatula*	F2H activities that has a catalytic mechanism similar to FNS II	[17]
	CYP93B16	soybean	direct conversion of flavanones into flavones	[18]
	FNSII-2.1	*Lonicera japonica*	direct conversion of flavanones into flavones	[61]
	FNSII-1.1	*Lonicera japonica*	direct conversion of flavanones into flavones	[61]
	FNSII-1.1	*Lonicera japonica*	direct conversion of flavanones into flavones	[61]
	CYP93B13		modulate the intensity of flower pigmentation	[62]
	CYP93B6		direct conversion of flavanones into flavones	[58]
CYP93C	CYP93C3	*Cicer arietinum*	no known	[63]
	CYP93C5	soybean	involved in isoflavone biosynthesis	[64]
	CYP93C1	soybean	involved in isoflavone biosynthesis	[64]
	CYP93C1v2	soybean	catalyzing the aryl migration	[65]
	CYP93C1v1	soybean	promoting nodulation gene expression in rhizobia	[66]
	CYP93C17	*Lotus japonicus*	involved in phytoalexin biosynthesis	[67]
	CYP93C2	*Glycyrrhiza echinata*	involved in isoflavonoid skeleton biosynthesis	[57]
	CYP93C18	*Pisum sativum*	involved in isoflavone phytoalexin, pisatin isoflavone phytoalexin, pisatin	[14,68]
	IFS2_12	*Trifolium repens*	involved in isoflavonoid genistein and its conjugates biosynthesis	[19]
CYP93E	CYP93E2	*Medicago truncatula*	involved in triterpenoid saponins biosynthesis	[69,23]
CYP93G	CYP93G3	sorghum	converting flavanones to flavone	[21]
	CYP93G5	maize	converts flavanones to 2-hydroxy derivatives served as substrates for C-glycosylation	[70]
	CYP93G1	Rice	channeling flavanones to the biosynthesis of tricin O-linked conjugates	[22]
	CYP93G2	Rice	convert naringenin and eriodictyol to the respective 2-hydroxyflavanones.	[20]
	CYP93G5	maize	a functional F2H under the genetic control of P1	[70–71]

Considering the functional conservation of CYP93B, CYP93C, and CYP93G homologous genes across different species (Table 1), as well as the high sequence conservation of CYP93 proteins in a given subfamily, although the roles of most CYP93 genes remain to be elucidated, we speculate that members of a subfamily might have recent common evolutionary origins and also a conserved function catalyzing the same metabolic reaction. For instance, flavones are ubiquitous secondary metabolites in plants; they are important compounds for the biochemistry and physiology of plants and human nutrition and health [51–54]. One of the two key flavone synthases, FNS I and FNS II, that catalyze the conversion of flavanones to flavones belong to the CYP93B subfamily [54–55]. To date, two subgroups have been characterized within the CYP93B gene family: one subgroup (FNS II) directly converts the flavanone substrates into flavones, such as CYP93B2 [56], CYP93B3 [57], and CYP93B6 [58]; conversely, the second (F2H) acts as flavanone 2-hydroxylases (CYP93B1, CYP93B10, and CYP93B11) that indirectly convert flavanone to flavones [59]. Consistent with the findings of these studies, our phylogenetic NJ tree results suggested that the flavanone 2-hydroxylases (F2Hs) are clustered into a clear clade separated from all other members of the CYP93B genes (FNS II; Fig 2). Interestingly, the monocot-specific clade included CYP93G subfamilies such as CYP93G1 and CYP93G2 in rice [20–22] and CYP93G3 in sorghum [21]; they share the same catalytic mechanism with the CYP93B subfamily by converting flavanones to flavones. Despite the large number of CYP93A genes as well as CYP93D and CYP93E genes that are embedded in or close to CYP93A genes, these genes were found to not contribute to flavonoid/isoflavonoid biosynthesis [50]; instead, the flavone synthase activity might have arisen after the evolution of CYP93B-K (Table 1). Thus, our results suggested that a CYP93 protein containing flavone synthase activity might be the ancestor of most of the present day CYP93 subfamilies, except CYP93A and CYP93E, which suggests monocot/eudicot divergence. Although the roles of CYP93D, CYP93F, CYP93H, CYP93J, and CYP93K remain to be elucidated, the CYP93 genes clustered in a subfamily shared similar gene architecture structure and functions, indicating recent common evolutionary origins and also conserved function. The knowledge of the functions of certain members should facilitate the confirmation of functional relationships among homologous genes.

Notably, despite the highly conserved sequence of CYP93 genes between subfamilies, significant functional specification exists. However, our selection analysis only revealed strong negative selection on the aligned coding region. Thus, the subfunctionalization of CYP93 gene family might not be derived from the mutation of active sites or active surrounding sites. In addition, we identified many subfamily-specific conserved insertions and deletions in some locations (Fig 3), implying a possible role in subfunctionalization. Moreover, considering the presence of many relaxed constraint sites in the less conserved region, they might also be important for the enzyme structure and contribute to the functional specification (Fig 3).

In addition, the expression pattern of a gene is often correlated with its function; the expression analysis might also provide important information regarding gene functions. Our expression analyses revealed that genes in the same subfamily across different species tend to have similar expression profiles (Figs 6−9), suggesting that the homologous CYP93 genes in a given subfamily share similar functions. For instance, most of the CYP93A genes have the same expression profile in the roots: CYP93C genes are mainly expressed in the roots. In *Arabidopsis* and soybean, CYP93 genes were expressed only or primarily in the roots, whereas, in monocots, they were expressed in many other tissues and organs (Figs 6 and 7). Hence, the common ancestor CYP93 might be widely expressed, but might be functional expressed only in the roots of some dicot plants such as *Arabidopsis*.

## Supporting Information

S1 Fig Phylogenetic tree of CYP93 proteins of the plant P450 superfamily. The maximum likelihood (ML) tree includes all of the 214 CYP93 candidate proteins used in the present study. Bootstrap values<50 are not shown.
(PDF)

S2 Fig Alignment of plant CYP93 proteins used in this study. MAFFT software was used for amino acid sequence alignment of the 214 CYP93 proteins. The shading of the alignment represents different degrees of conservation among sequences. The P450 motifs and substrate recognition sites are underlined.
(PDF)

S3 Fig Modeled structure of CYP93 of each group. The overall structures of CYP93 are very similar. Red spheres represent conserved insertion of B1 and C. The model was built using SWISS-MODEL (http://swissmodel.expasy.org/) and viewed using PyMOL software.
(PDF)

S4 Fig Phylogenetic tree of CYP93 proteins and representative of each family of the plant P450 superfamily. The neighbor-joining (NJ) tree includes the the representatives of the 10 CYP93 subfamilies and 57 representatives of other plant P450 families. Bootstrap values <50 are not shown. CYP51 is the root of the tree.
(PDF)

S1 Table The query sequences used in the identification of CYP93 proteins in each species. (XLS)

S2 Table The species used in this study and the corresponding CYP93 candidate genes identified. (XLSX)

S3 Table Primers used in this study. (XLSX)

S4 Table The identity and similarity analysis of plant CYP93 proteins. (XLSX)

S5 Table The selection analysis of different branches of CYP93 gene family. (XLSX)
