# Supplementary material for: Genome-Wide Analysis, Classification, Evolution, and Expression Analysis of the Cytochrome P450 93 Family in Land Plants
Source: PLoS One. 2016 Oct 19;11(10):e0165020. doi: 10.1371/journal.pone.0165020 (PMC5070762; doi:10.1371/journal.pone.0165020)
Supplement: S1 Fig — The maximum likelihood (ML) tree includes all of the 214 CYP93 candidate proteins used in the present study. Bootstrap values<50 are not shown. (PDF) [file pone.0165020.s001.pdf]

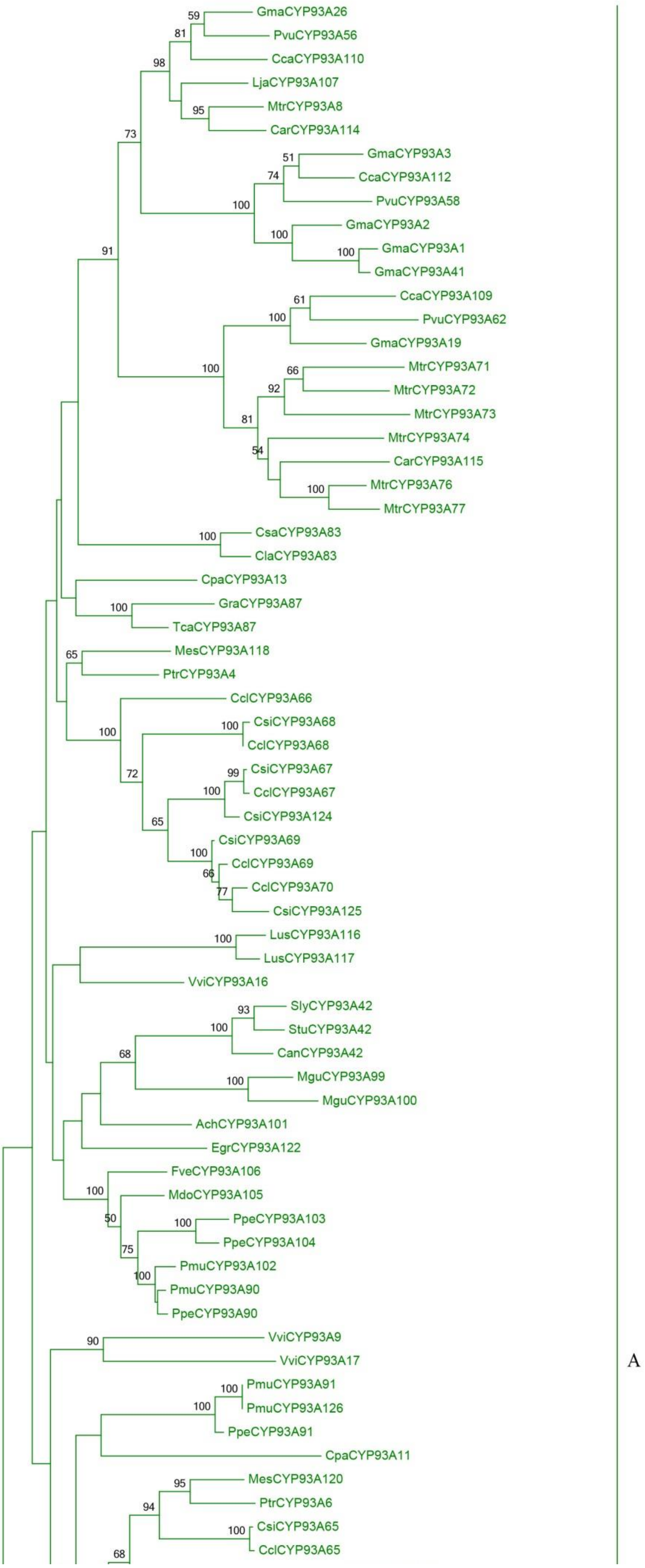
GmaCYP93A26
PvuCYP93A56
CcaCYP93A110
LjaCYP93A107
MtrCYP93A8
CarCYP93A114
GmaCYP93A3
CcaCYP93A112
PvuCYP93A58
GmaCYP93A2
GmaCYP93A1
GmaCYP93A41
CcaCYP93A109
PvuCYP93A62
GmaCYP93A19
MtrCYP93A71
MtrCYP93A72
MtrCYP93A73
MtrCYP93A74
CarCYP93A115
MtrCYP93A76
MtrCYP93A77
CsaCYP93A83
ClaCYP93A83
CpaCYP93A13
GraCYP93A87
TcaCYP93A87
MesCYP93A118
PtrCYP93A4
CclCYP93A66
CsiCYP93A68
CclCYP93A68
CsiCYP93A67
CclCYP93A67
CsiCYP93A124
CsiCYP93A69
CclCYP93A69
CclCYP93A70
CsiCYP93A125
LusCYP93A116
LusCYP93A117
VviCYP93A16
SlyCYP93A42
StuCYP93A42
CanCYP93A42
MguCYP93A99
MguCYP93A100
AchCYP93A101
EgrCYP93A122
FveCYP93A106
MdoCYP93A105
PpeCYP93A103
PpeCYP93A104
PmuCYP93A102
PmuCYP93A90
PpeCYP93A90
VviCYP93A9
VviCYP93A17
PmuCYP93A91
PmuCYP93A126
PpeCYP93A91
CpaCYP93A11
MesCYP93A120
PtrCYP93A6
CsiCYP93A65
CclCYP93A65
A

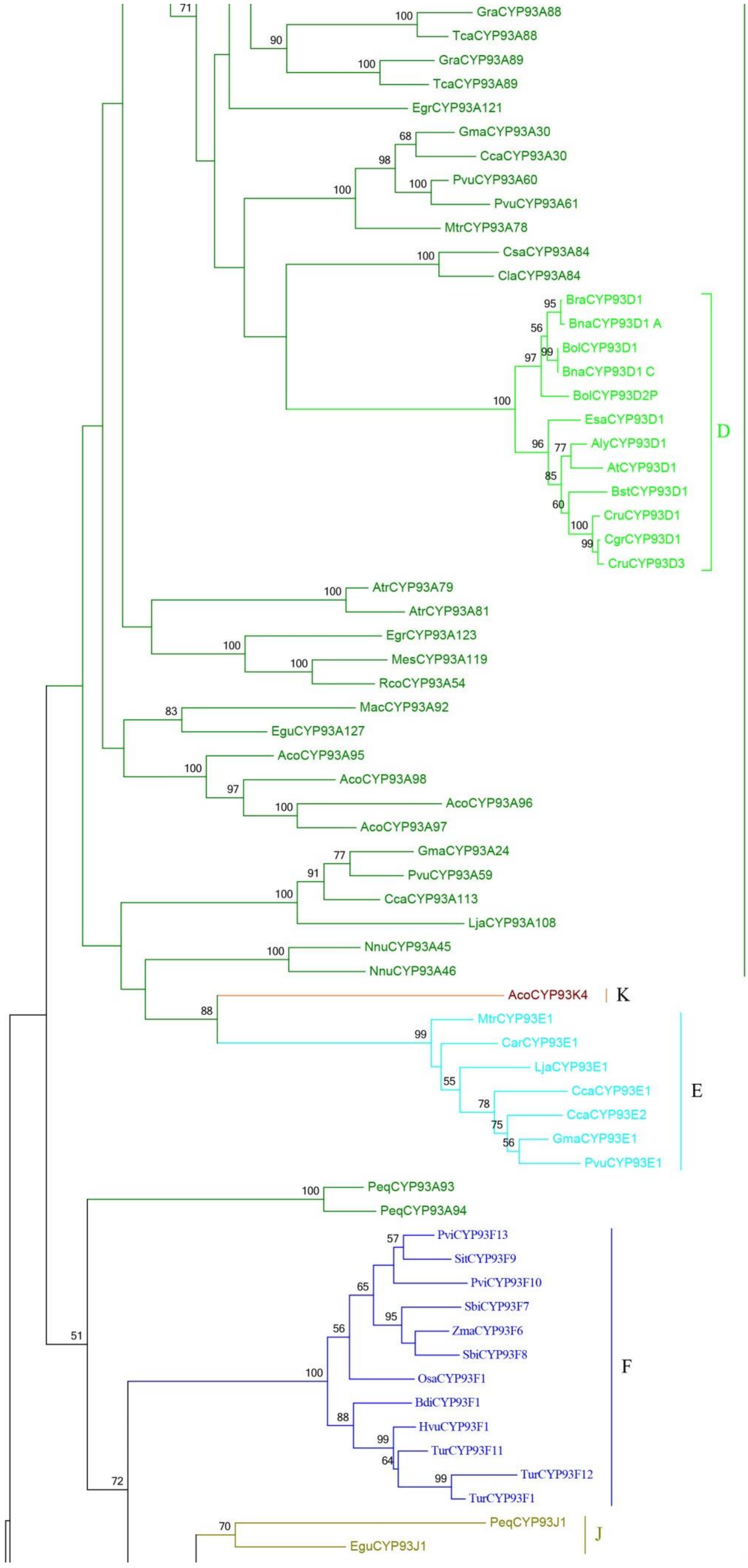

GraCYP93A88
TcaCYP93A88
GraCYP93A89
TcaCYP93A89
EgrCYP93A121
GmaCYP93A30
CcaCYP93A30
PvuCYP93A60
PvuCYP93A61
MtrCYP93A78
CsaCYP93A84
ClaCYP93A84
BraCYP93D1
BnaCYP93D1 A
BolCYP93D1
BnaCYP93D1 C
BolCYP93D2P
EsaCYP93D1
AlyCYP93D1
AtCYP93D1
BstCYP93D1
CruCYP93D1
CgrCYP93D1
CruCYP93D3
D
AtrCYP93A79
AtrCYP93A81
EgrCYP93A123
MesCYP93A119
RcoCYP93A54
MacCYP93A92
EguCYP93A127
AcoCYP93A95
AcoCYP93A98
AcoCYP93A96
AcoCYP93A97
GmaCYP93A24
PvuCYP93A59
CcaCYP93A113
LjaCYP93A108
NnuCYP93A45
NnuCYP93A46
AcoCYP93K4
K
MtrCYP93E1
CarCYP93E1
LjaCYP93E1
CcaCYP93E1
CcaCYP93E2
GmaCYP93E1
PvuCYP93E1
E
PeqCYP93A93
PeqCYP93A94
PviCYP93F13
SitCYP93F9
PviCYP93F10
SbiCYP93F7
ZmaCYP93F6
SbiCYP93F8
OsaCYP93F1
BdiCYP93F1
HvuCYP93F1
TurCYP93F11
TurCYP93F12
TurCYP93F1
F
PeqCYP93J1
EguCYP93J1
J

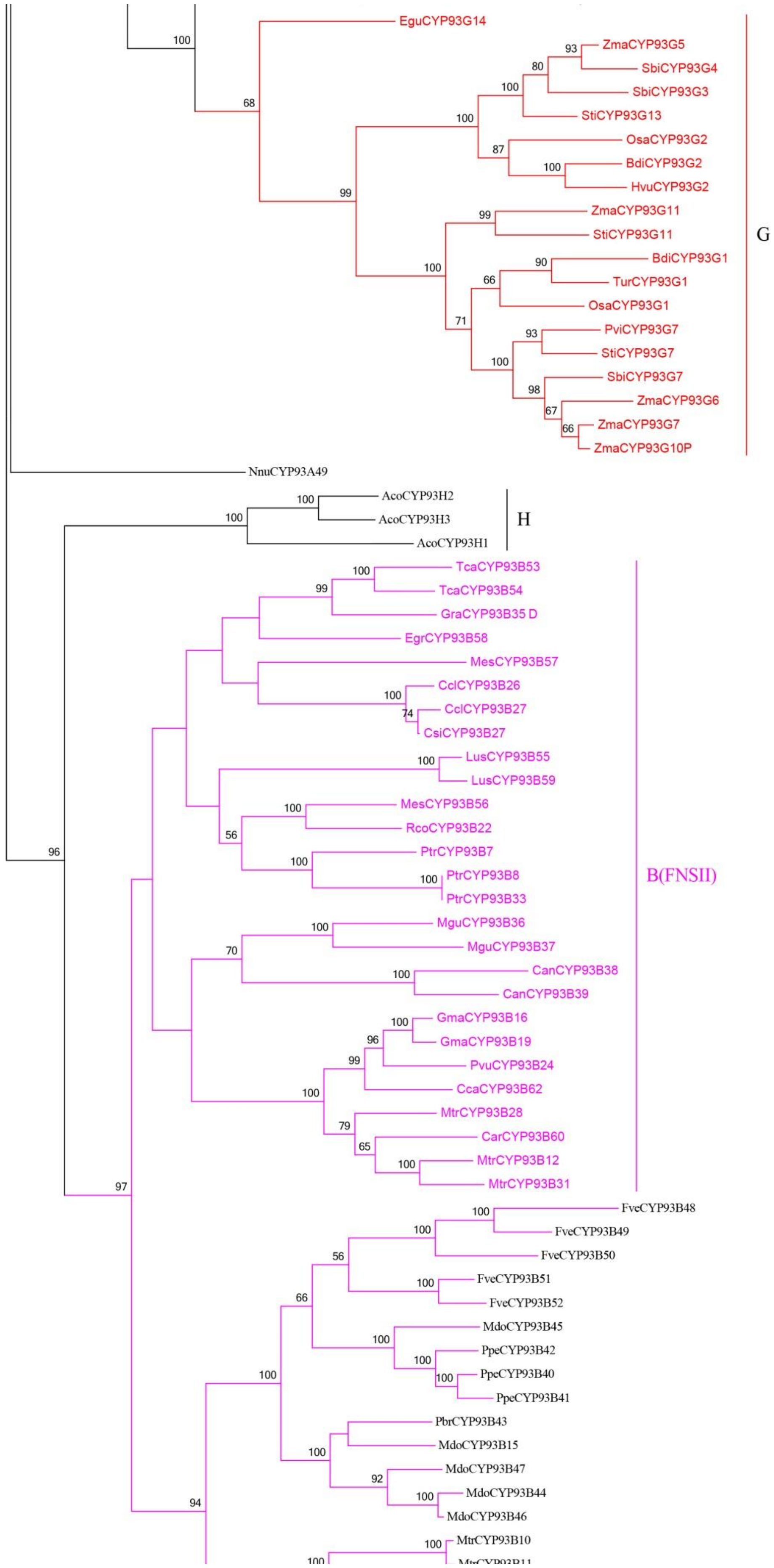
EguCYP93G14
ZmaCYP93G5
SbiCYP93G4
SbiCYP93G3
StiCYP93G13
OsaCYP93G2
BdiCYP93G2
HvuCYP93G2
ZmaCYP93G11
StiCYP93G11
BdiCYP93G1
TurCYP93G1
OsaCYP93G1
PviCYP93G7
StiCYP93G7
SbiCYP93G7
ZmaCYP93G6
ZmaCYP93G7
ZmaCYP93G10P
G
NnuCYP93A49
AcoCYP93H2
AcoCYP93H3
AcoCYP93H1
H
TcaCYP93B53
TcaCYP93B54
GraCYP93B35 D
EgrCYP93B58
MesCYP93B57
CclCYP93B26
CclCYP93B27
CsiCYP93B27
LusCYP93B55
LusCYP93B59
MesCYP93B56
RcoCYP93B22
PtrCYP93B7
PtrCYP93B8
PtrCYP93B33
B(FNSII)
MguCYP93B36
MguCYP93B37
CanCYP93B38
CanCYP93B39
GmaCYP93B16
GmaCYP93B19
PvuCYP93B24
CcaCYP93B62
MtrCYP93B28
CarCYP93B60
MtrCYP93B12
MtrCYP93B31
FveCYP93B48
FveCYP93B49
FveCYP93B50
FveCYP93B51
FveCYP93B52
MdoCYP93B45
PpeCYP93B42
PpeCYP93B40
PpeCYP93B41
PbrCYP93B43
MdoCYP93B15
MdoCYP93B47
MdoCYP93B44
MdoCYP93B46
MtrCYP93B10

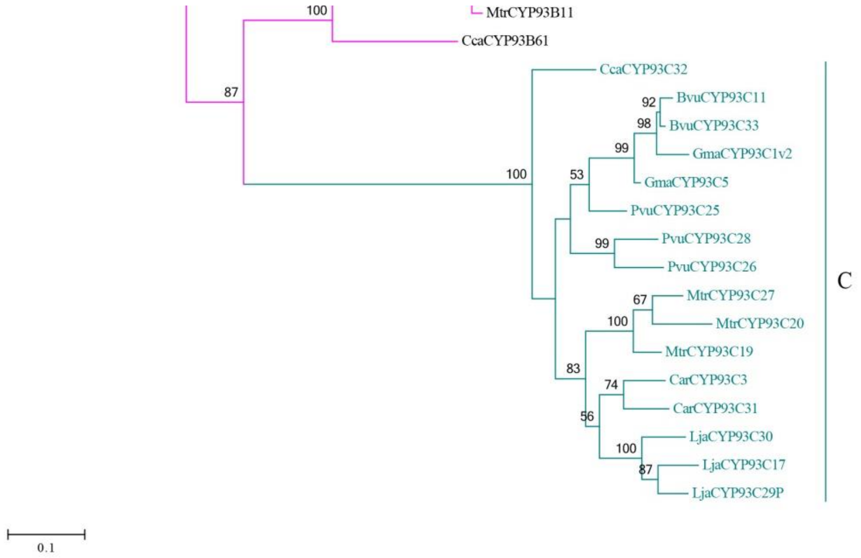

S4 Fig. Phylogenetic tree of CYP93 proteins and representative of each family of the plant P450 superfamily. The neighbor-joining (NJ) tree includes the the representatives of the 10 CYP93 subfamilies and 57 representatives of other plant P450 families. Bootstrap values of <50 are not shown. CYP51 is the root of the tree.
