# Supplementary material for: Genome-Wide Analysis, Classification, Evolution, and Expression Analysis of the Cytochrome P450 93 Family in Land Plants
Source: PLoS One. 2016 Oct 19;11(10):e0165020. doi: 10.1371/journal.pone.0165020 (PMC5070762; doi:10.1371/journal.pone.0165020)
Supplement: S2 Fig — The shading of the alignment represents different degrees of conservation among sequences. The P450 motifs and substrate recognition sites are underlined. (PDF) [file pone.0165020.s002.pdf]

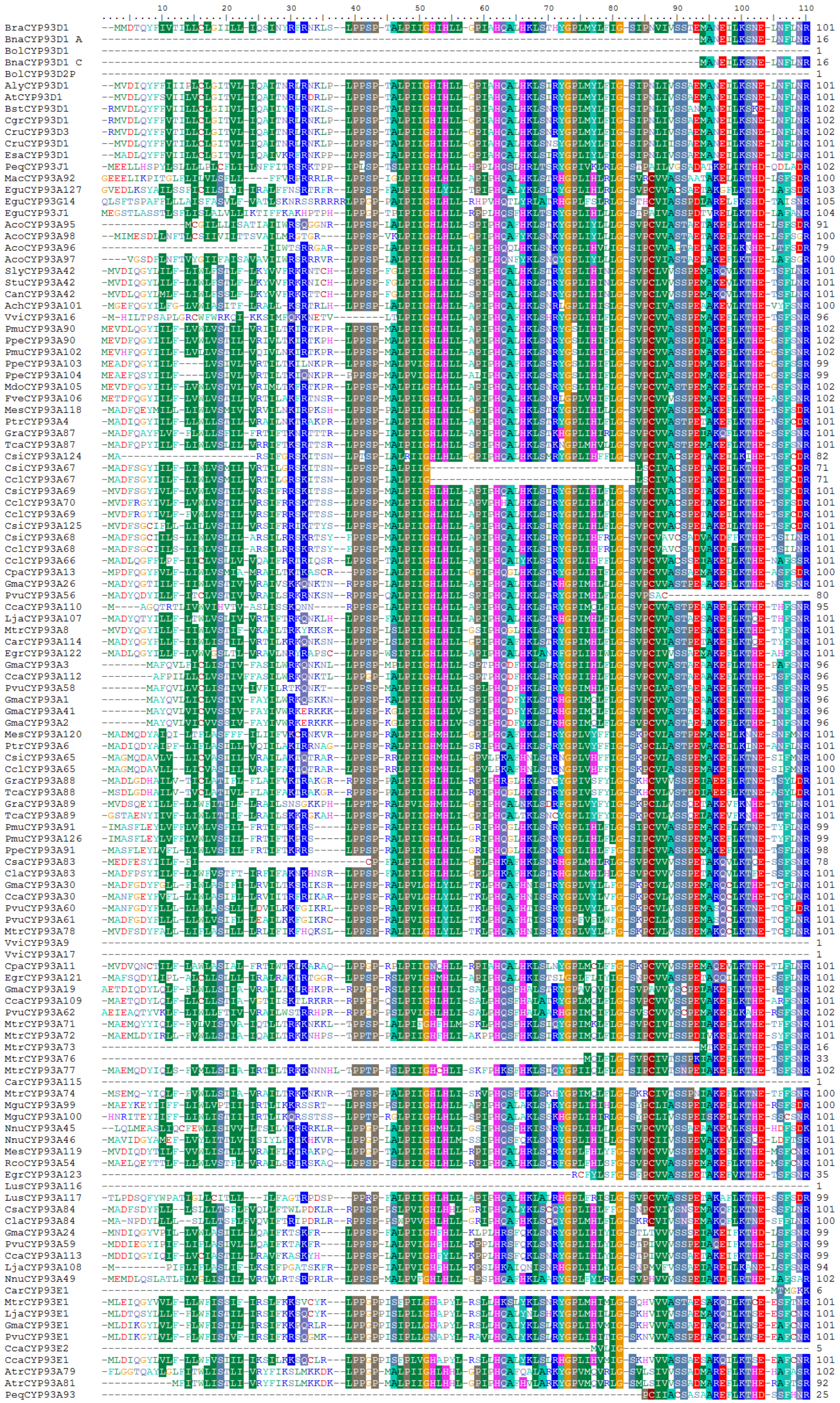
BraCYP93D1
BnaCYP93D1 A
BolCYP93D1
BnaCYP93D1 C
BolCYP93D2P
AlyCYP93D1
AtCYP93D1
BstCYP93D1
CgrCYP93D1
CruCYP93D3
CruCYP93D1
EsaCYP93D1
PeqCYP93J1
MacCYP93A92
EguCYP93A127
EguCYP93G14
EguCYP93J1
AcoCYP93A95
AcoCYP93A98
AcoCYP93A96
AcoCYP93A97
SlyCYP93A42
StuCYP93A42
CanCYP93A42
AchCYP93A101
VviCYP93A16
PmuCYP93A90
PpeCYP93A90
PmuCYP93A102
PpeCYP93A103
PpeCYP93A104
MdoCYP93A105
FveCYP93A106
MesCYP93A118
PtrCYP93A4
GraCYP93A87
TcaCYP93A87
CsiCYP93A124
CsiCYP93A67
CclCYP93A67
CsiCYP93A69
CclCYP93A70
CclCYP93A69
CsiCYP93A125
CsiCYP93A68
CclCYP93A68
CclCYP93A66
CpaCYP93A13
GmaCYP93A26
PvuCYP93A56
CcaCYP93A110
LjaCYP93A107
MtrCYP93A8
CarCYP93A114
EgrCYP93A122
GmaCYP93A3
CcaCYP93A112
PvuCYP93A58
GmaCYP93A1
GmaCYP93A41
GmaCYP93A2
MesCYP93A120
PtrCYP93A6
CsiCYP93A65
CclCYP93A65
GraCYP93A88
TcaCYP93A88
GraCYP93A89
TcaCYP93A89
PmuCYP93A91
PmuCYP93A126
PpeCYP93A91
CsaCYP93A83
ClaCYP93A83
GmaCYP93A30
CcaCYP93A30
PvuCYP93A60
PvuCYP93A61
MtrCYP93A78
VviCYP93A9
VviCYP93A17
CpaCYP93A11
EgrCYP93A121
GmaCYP93A19
CcaCYP93A109
PvuCYP93A62
MtrCYP93A71
MtrCYP93A72
MtrCYP93A73
MtrCYP93A76
MtrCYP93A77
CarCYP93A115
MtrCYP93A74
MguCYP93A99
MguCYP93A100
NnuCYP93A45
NnuCYP93A46
MesCYP93A119
RcoCYP93A54
EgrCYP93A123
LusCYP93A116
LusCYP93A117
CsaCYP93A84
ClaCYP93A84
GmaCYP93A24
PvuCYP93A59
CcaCYP93A113
LjaCYP93A108
NnuCYP93A49
CarCYP93E1
MtrCYP93E1
LjaCYP93E1
GmaCYP93E1
PvuCYP93E1
CcaCYP93E2
CcaCYP93E1
AtrCYP93A79
AtrCYP93A81
PeqCYP93A93

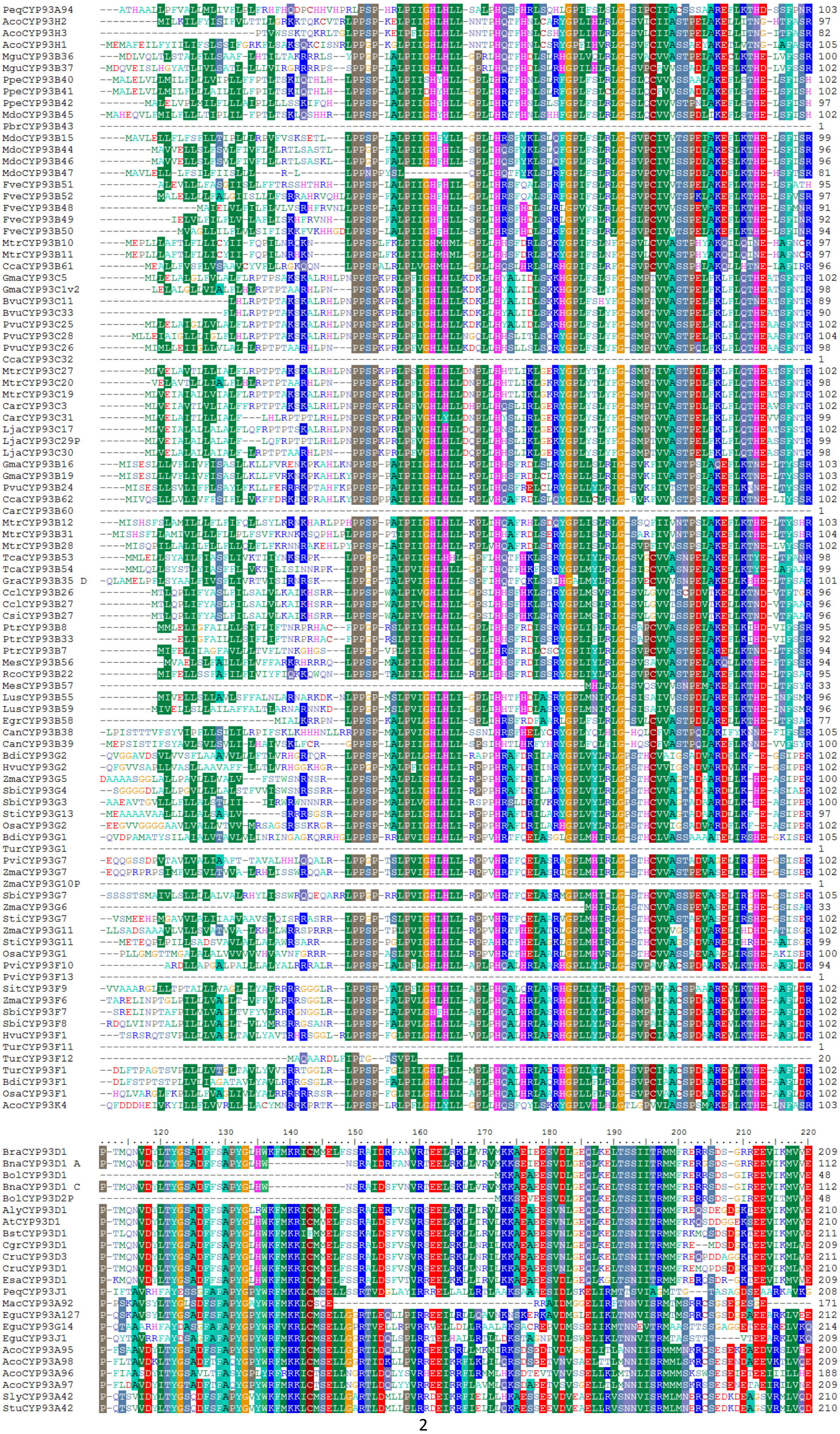

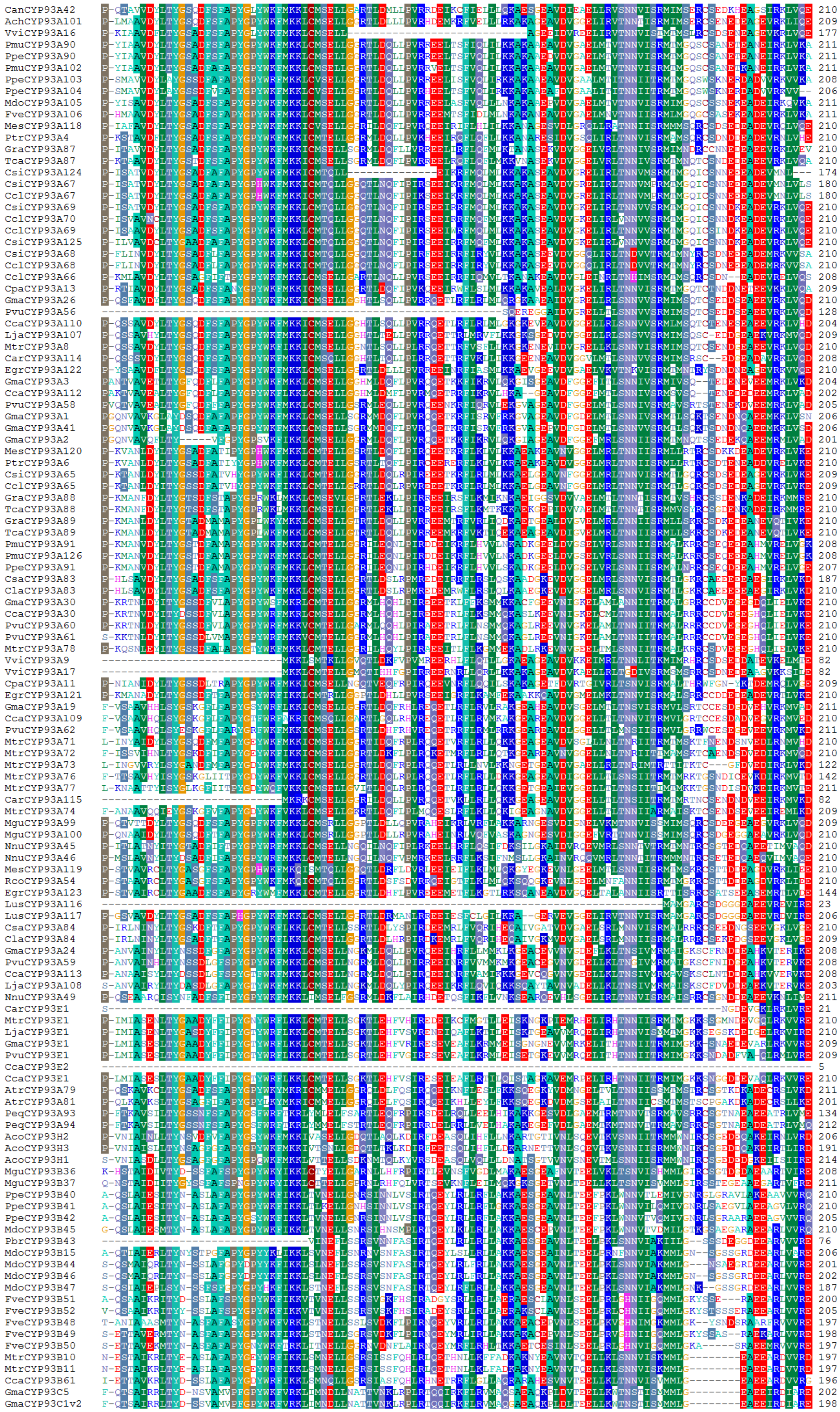
CanCYP93A42
AchCYP93A101
VviCYP93A16
PmuCYP93A90
PpeCYP93A90
PmuCYP93A102
PpeCYP93A103
PpeCYP93A104
MdoCYP93A105
FveCYP93A106
MesCYP93A118
PtrCYP93A4
GraCYP93A87
TcaCYP93A87
CsiCYP93A124
CsiCYP93A67
CclCYP93A67
CsiCYP93A69
CclCYP93A70
CclCYP93A69
CsiCYP93A125
CsiCYP93A68
CclCYP93A68
CclCYP93A66
CpaCYP93A13
GmaCYP93A26
PvuCYP93A56
CcaCYP93A110
LjaCYP93A107
MtrCYP93A8
CarCYP93A114
EgrCYP93A122
GmaCYP93A3
CcaCYP93A112
PvuCYP93A58
GmaCYP93A1
GmaCYP93A41
GmaCYP93A2
MesCYP93A120
PtrCYP93A6
CsiCYP93A65
CclCYP93A65
GraCYP93A88
TcaCYP93A88
GraCYP93A89
TcaCYP93A89
PmuCYP93A91
PmuCYP93A126
PpeCYP93A91
CsaCYP93A83
ClaCYP93A83
GmaCYP93A30
CcaCYP93A30
PvuCYP93A60
PvuCYP93A61
MtrCYP93A78
VviCYP93A9
VviCYP93A17
CpaCYP93A11
EgrCYP93A121
GmaCYP93A19
CcaCYP93A109
PvuCYP93A62
MtrCYP93A71
MtrCYP93A72
MtrCYP93A73
MtrCYP93A76
MtrCYP93A77
CarCYP93A115
MtrCYP93A74
MguCYP93A99
MguCYP93A100
NnuCYP93A45
NnuCYP93A46
MesCYP93A119
RcoCYP93A54
EgrCYP93A123
LusCYP93A116
LusCYP93A117
CsaCYP93A84
ClaCYP93A84
GmaCYP93A24
PvuCYP93A59
CcaCYP93A113
LjaCYP93A108
NnuCYP93A49
CarCYP93E1
MtrCYP93E1
LjaCYP93E1
GmaCYP93E1
PvuCYP93E1
CcaCYP93E2
CcaCYP93E1
AtrCYP93A79
AtrCYP93A81
PeqCYP93A93
PeqCYP93A94
AcoCYP93H2
AcoCYP93H3
AcoCYP93H1
MguCYP93B36
MguCYP93B37
PpeCYP93B40
PpeCYP93B41
PpeCYP93B42
MdoCYP93B45
PbrCYP93B43
MdoCYP93B15
MdoCYP93B44
MdoCYP93B46
MdoCYP93B47
FveCYP93B51
FveCYP93B52
FveCYP93B48
FveCYP93B49
FveCYP93B50
MtrCYP93B10
MtrCYP93B11
CcaCYP93B61
GmaCYP93C5
GmaCYP93C1v2

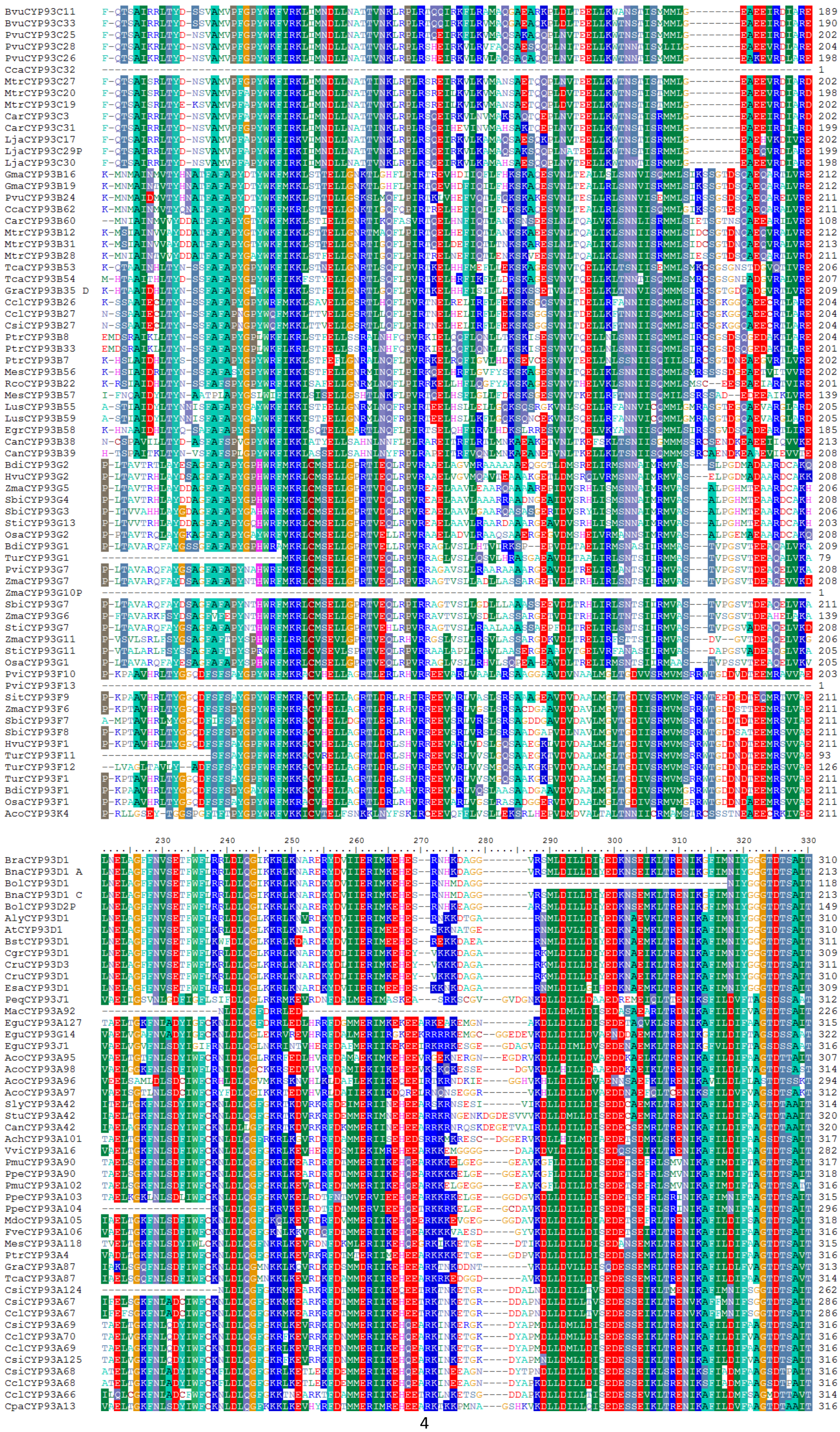

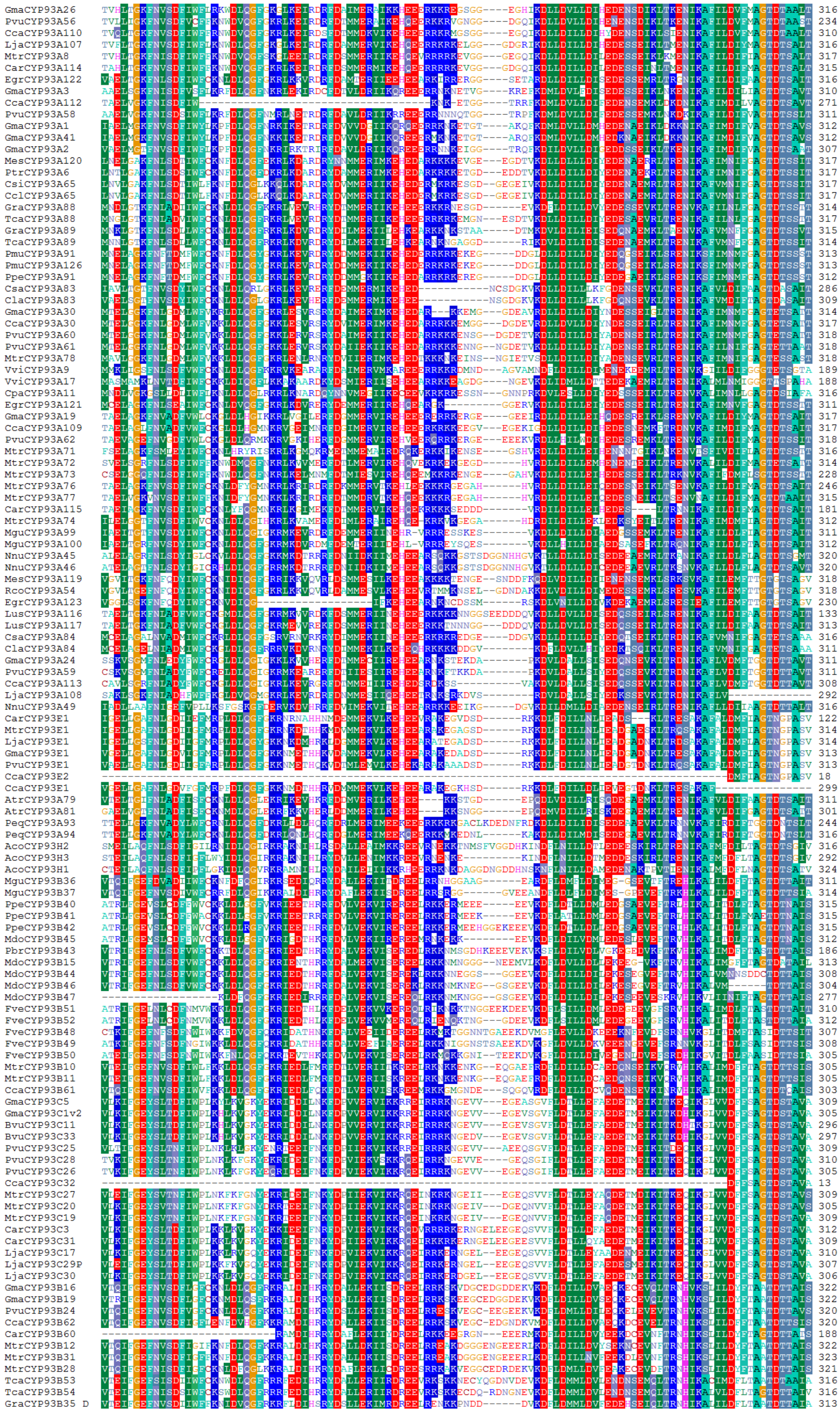

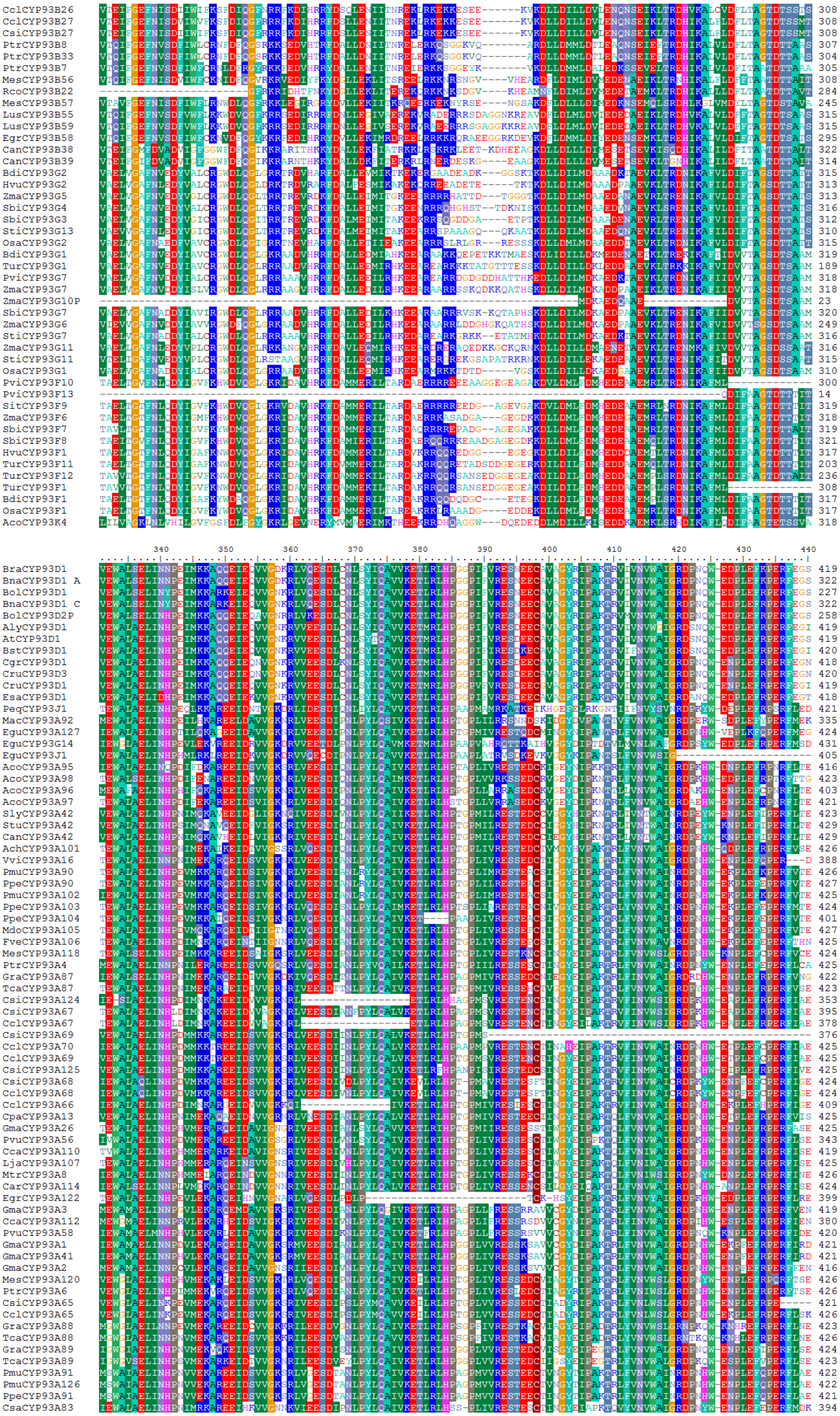

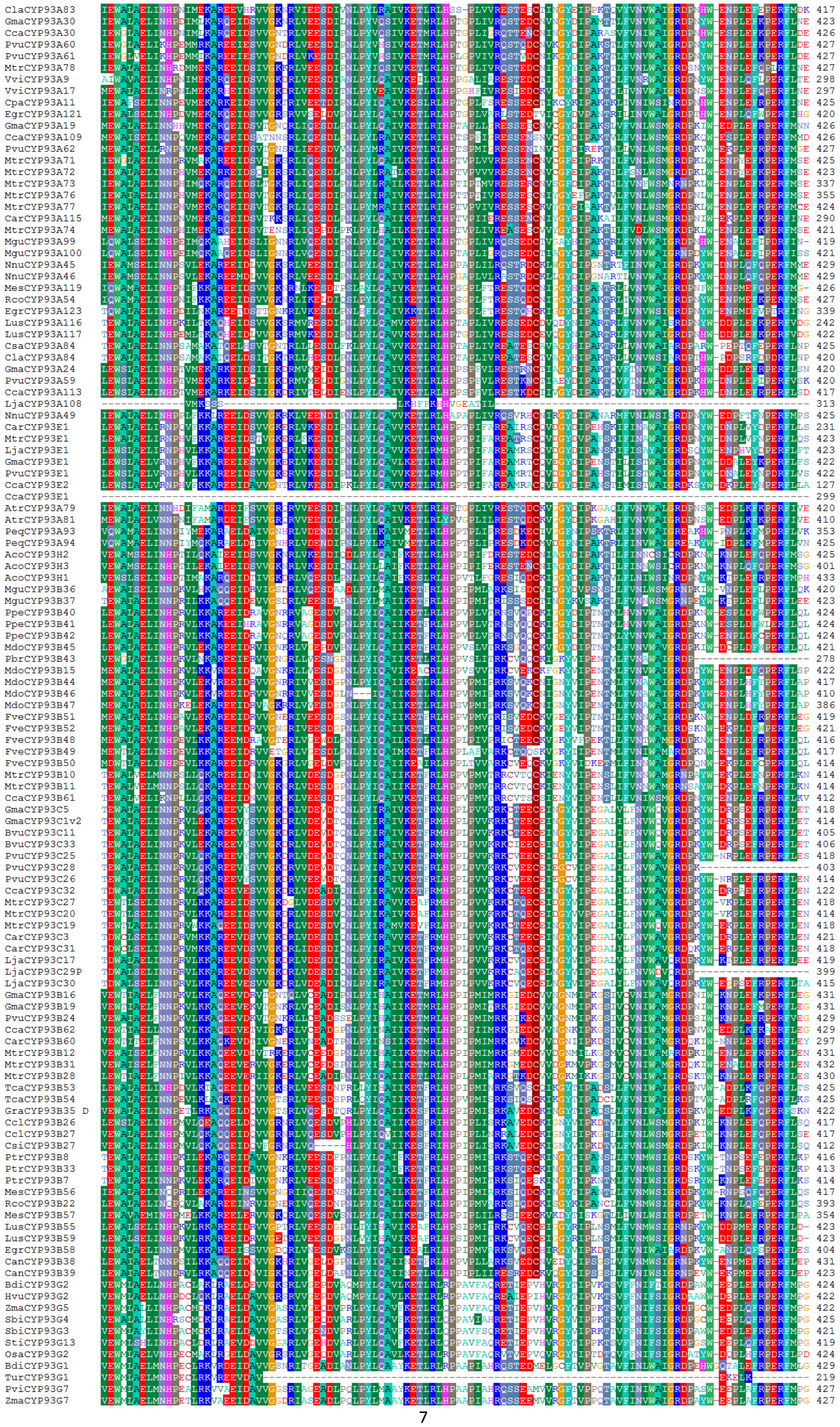

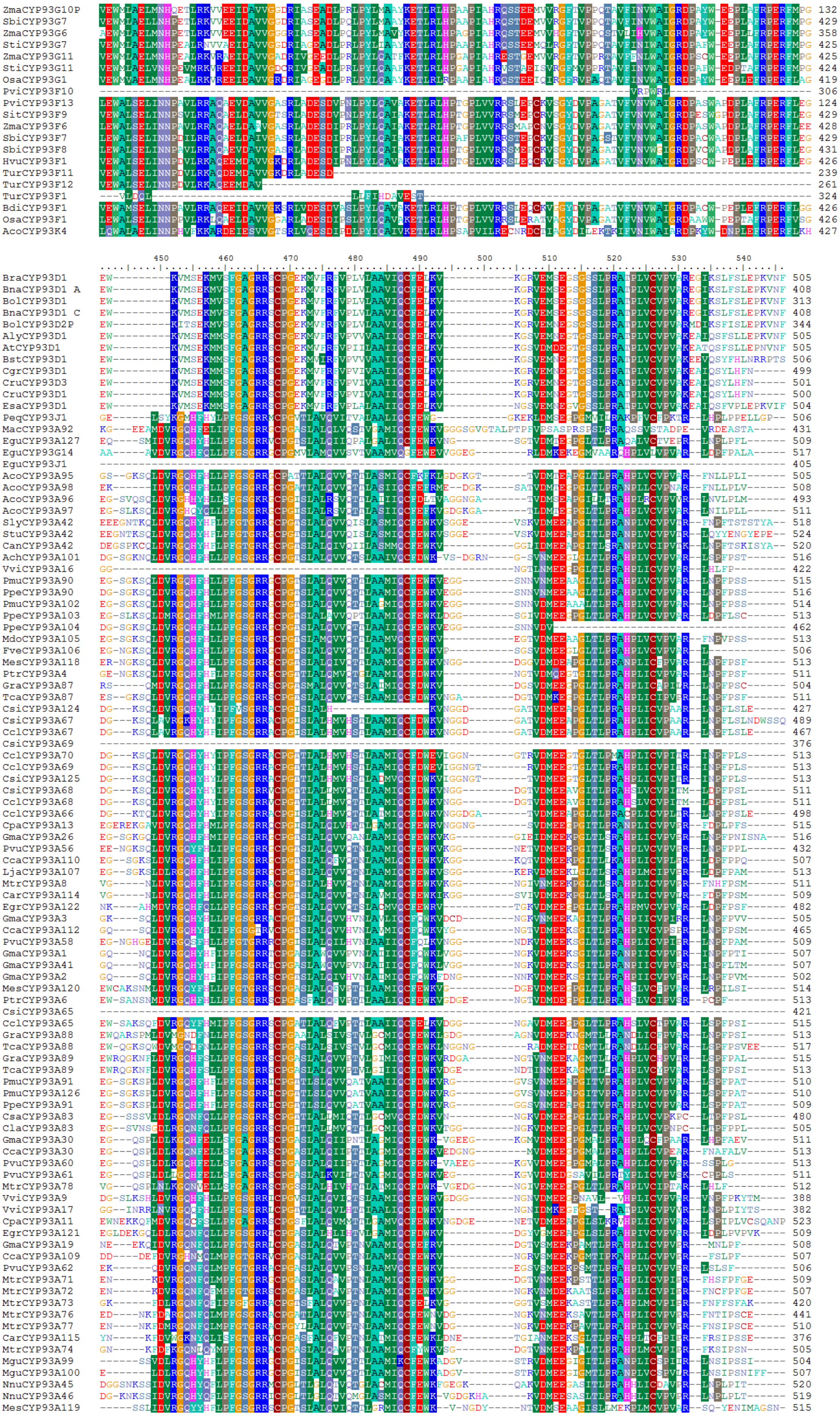

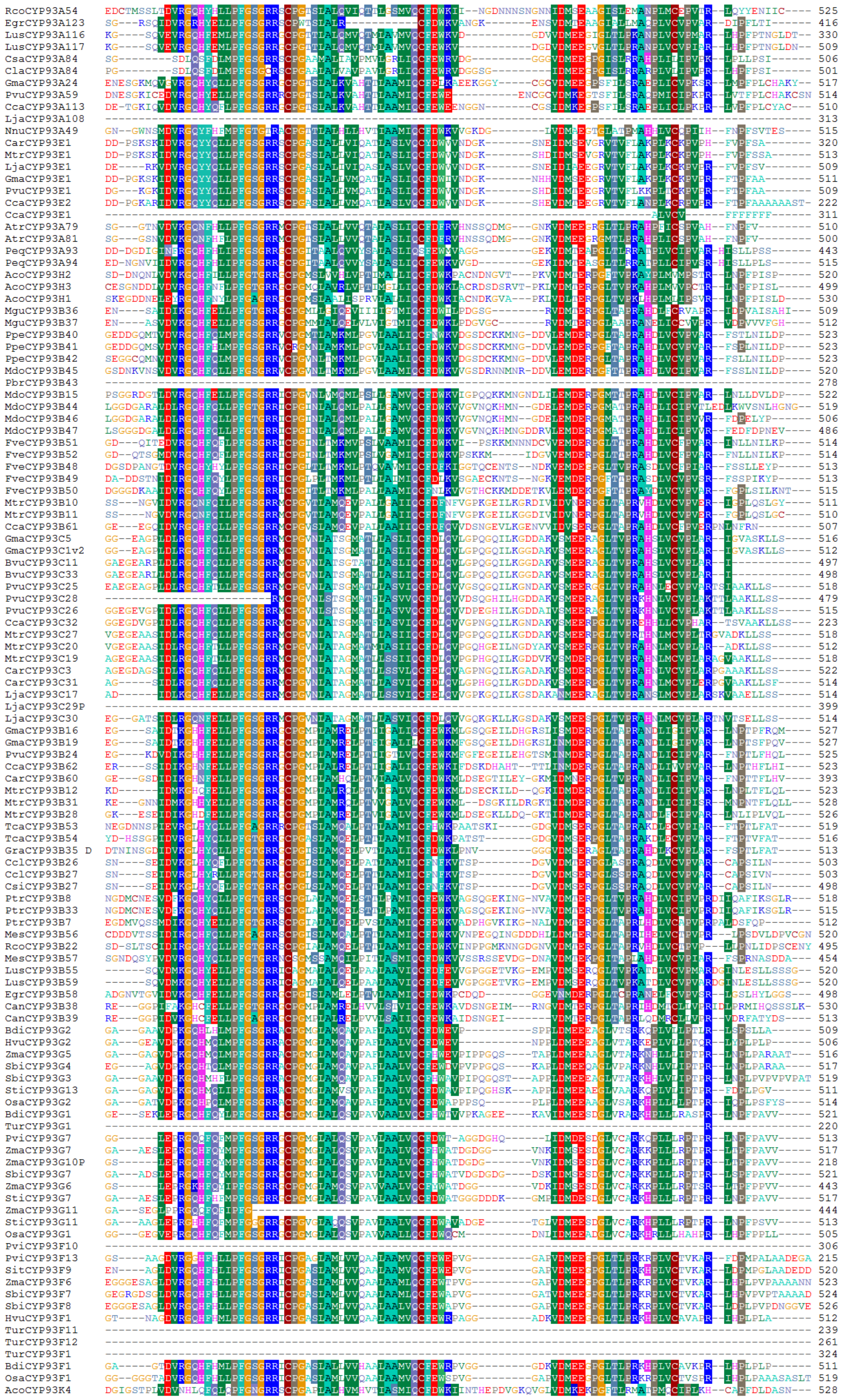
RcoCYP93A54
EgrCYP93A123
LusCYP93A116
LusCYP93A117
CsaCYP93A84
ClaCYP93A84
GmaCYP93A24
PvuCYP93A59
CcaCYP93A113
LjaCYP93A108
NnuCYP93A49
CarCYP93E1
MtrCYP93E1
LjaCYP93E1
GmaCYP93E1
PvuCYP93E1
CcaCYP93E2
CcaCYP93E1
AtrCYP93A79
AtrCYP93A81
PegCYP93A93
PegCYP93A94
AcoCYP93H2
AcoCYP93H3
AcoCYP93H1
MguCYP93B36
MguCYP93B37
PpeCYP93B40
PpeCYP93B41
PpeCYP93B42
MdoCYP93B45
PbrCYP93B43
MdoCYP93B15
MdoCYP93B44
MdoCYP93B46
MdoCYP93B47
FveCYP93B51
FveCYP93B52
FveCYP93B48
FveCYP93B49
FveCYP93B50
MtrCYP93B10
MtrCYP93B11
CcaCYP93B61
GmaCYP93C5
GmaCYP93C1v2
BvuCYP93C11
BvuCYP93C33
PvuCYP93C25
PvuCYP93C28
PvuCYP93C26
CcaCYP93C32
MtrCYP93C27
MtrCYP93C20
MtrCYP93C19
CarCYP93C3
CarCYP93C31
LjaCYP93C17
LjaCYP93C29P
LjaCYP93C30
GmaCYP93B16
GmaCYP93B19
PvuCYP93B24
CcaCYP93B62
CarCYP93B60
MtrCYP93B12
MtrCYP93B31
MtrCYP93B28
TcaCYP93B53
TcaCYP93B54
GraCYP93B35 D
CclCYP93B26
CclCYP93B27
CsiCYP93B27
PtrCYP93B8
PtrCYP93B33
PtrCYP93B7
MesCYP93B56
RcoCYP93B22
MesCYP93B57
LusCYP93B55
LusCYP93B59
EgrCYP93B58
CanCYP93B38
CanCYP93B39
BdiCYP93G2
HvuCYP93G2
ZmaCYP93G5
SbiCYP93G4
SbiCYP93G3
StiCYP93G13
OsaCYP93G2
BdiCYP93G1
TurCYP93G1
PviCYP93G7
ZmaCYP93G7
ZmaCYP93G10P
SbiCYP93G7
ZmaCYP93G6
StiCYP93G7
ZmaCYP93G11
StiCYP93G11
OsaCYP93G1
PviCYP93F10
PviCYP93F13
SitCYP93F9
ZmaCYP93F6
SbiCYP93F7
SbiCYP93F8
HvuCYP93F1
TurCYP93F11
TurCYP93F12
TurCYP93F1
BdiCYP93F1
OsaCYP93F1
AcoCYP93K4

S2 Fig. Alignment of the plant CYP93 proteins used in this study. MAFFT software was used to apply thefor amino acid sequence alignment of the 214 CYP93 proteins. The shading of the alignment represents different degrees of conservation among sequences. The P450 motifs and substrate recognition sites are underlined.
