# Supplementary material for: Genome-Wide Analysis, Classification, Evolution, and Expression Analysis of the Cytochrome P450 93 Family in Land Plants
Source: PLoS One. 2016 Oct 19;11(10):e0165020. doi: 10.1371/journal.pone.0165020 (PMC5070762; doi:10.1371/journal.pone.0165020)
Supplement: S3 Fig — The overall structures of CYP93 are very similar. Red spheres represent conserved insertion of B1 and C. The model was built using SWISS-MODEL (http://swissmodel.expasy.org/) and viewed using PyMOL software. (PDF) [file pone.0165020.s003.pdf]

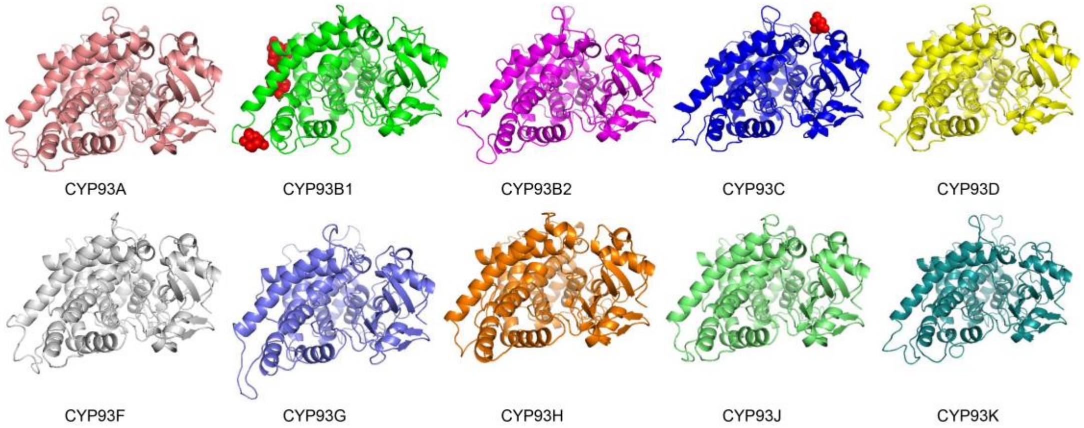

S3 Fig: Modeled structure of CYP93 of each group. The overall structures of CYP93 are very similar. Red spheres represent conserved insertion of B1 and C. The model was built using SWISS-MODEL (http://swissmodel.expasy.org/) and viewed using PyMOL software.
