# Supplementary material for: Genome-Wide Analysis, Classification, Evolution, and Expression Analysis of the Cytochrome P450 93 Family in Land Plants
Source: PLoS One. 2016 Oct 19;11(10):e0165020. doi: 10.1371/journal.pone.0165020 (PMC5070762; doi:10.1371/journal.pone.0165020)
Supplement: S4 Fig — The neighbor-joining (NJ) tree includes the the representatives of the 10 CYP93 subfamilies and 57 representatives of other plant P450 families. Bootstrap values <50 are not shown. CYP51 is the root of the tree. (PDF) [file pone.0165020.s004.pdf]

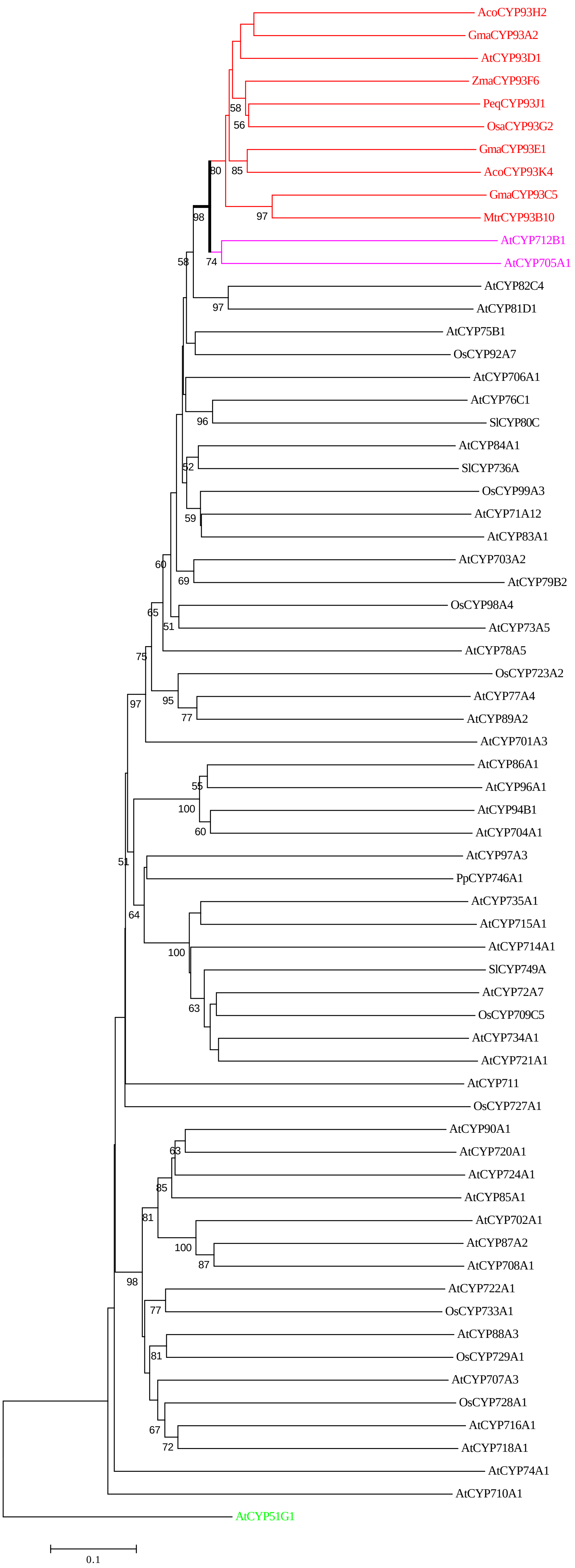

S4 Fig. Phylogenetic tree of CYP93 proteins and representative of each family of the plant P450 superfamily. The neighbor-joining (NJ) tree includes the the representatives of the 10 CYP93 subfamilies and 57 representatives of other plant P450 families. Bootstrap values of <50 are not shown. CYP51 is the root of the tree.
